# Integrated Metabolomics and Transcriptomics Analysis of Anacardic Acid Inhibition of Breast Cancer Cell Viability

**DOI:** 10.3390/ijms25137044

**Published:** 2024-06-27

**Authors:** Kellianne M. Piell, Claire C. Poulton, Christian G. Stanley, David J. Schultz, Carolyn M. Klinge

**Affiliations:** 1 Department of Biochemistry & Molecular Genetics, University of Louisville School of Medicine, Louisville, KY 40292, USA; 2 Department of Biology, University of Louisville, Louisville, KY 40292, USA

**Keywords:** anacardic acid, breast cancer, TNBC, metabolomics, transcriptomics

## Abstract

Anacardic acid (AnAc) inhibits the growth of estrogen receptor α (ERα)-positive MCF-7 breast cancer (BC) cells and MDA-MB-231 triple-negative BC (TNBC) cells, without affecting primary breast epithelial cells. RNA sequencing (seq) and network analysis of AnAc-treated MCF-7 and MDA-MB-231 cells suggested that AnAc inhibited lipid biosynthesis and increased endoplasmic reticulum stress. To investigate the impact of AnAc on cellular metabolism, a comprehensive untargeted metabolomics analysis was performed in five independent replicates of control versus AnAc-treated MCF-7 and MDA-MB-231 cells and additional TNBC cell lines: MDA-MB-468, BT-20, and HCC1806. An analysis of the global metabolome identified key metabolic differences between control and AnAc-treated within each BC cell line and between MCF-7 and the TNBC cell lines as well as metabolic diversity among the four TNBC cell lines, reflecting TNBC heterogeneity. AnAc-regulated metabolites were involved in alanine, aspartate, glutamate, and glutathione metabolism; the pentose phosphate pathway; and the citric acid cycle. Integration of the transcriptome and metabolome data for MCF-7 and MDA-MB-231 identified Signal transduction: mTORC1 downstream signaling in both cell lines and additional cell-specific pathways. Together, these data suggest that AnAc treatment differentially alters multiple pools of cellular building blocks, nutrients, and transcripts resulting in reduced BC cell viability.

## 1. Introduction

Breast cancer (BC) is the second leading cause of cancer death in U.S. women [1] and worldwide [2]. Most breast tumors express estrogen receptor α (ER) and progesterone receptor (PR), and patients receive adjuvant endocrine therapies depending on menopausal status, namely tamoxifen (premenopausal) or aromatase inhibitors (postmenopausal), with CDK4/6, PI3K, and mTOR inhibitors for metastatic disease [3]. Approximately 10–15% of all female breast tumors are classified as triple-negative BC (TNBC) [4]. TNBC tumors lack ER, PR, and human epidermal growth factor receptor 2, (ERBB2, HER2) proteins which are successfully targeted by clinical adjuvant therapies [5]. Instead, patients with TNBC receive chemotherapy, PARP (Poly(ADP-ribose) Polymerase) inhibitors, i.e., olaparib and talazoparib, and antibody–drug conjugates (ADCs, i.e., sacituzumab govitecan) [6]. Patients with TNBC have lower relapse-free and overall survival (RFS and OS, respectively) [7]. TNBC tumors are heterogeneous and include basal-like type 1 (BL1), basal-like type 2 (BL2), mesenchymal (M), mesenchymal stem-like (MLS), and luminal androgen receptor (LAR) subtypes [8,9].

Metabolic dysregulation is a feature of TNBC that contributes to metastasis and offers new therapeutic targets [10,11]. TNBC was subdivided into three heterogeneous metabolic pathway-based subtypes (MPS1, MPS2, and MPS3) based on transcriptomic profiling [12]. MPS1 has upregulated lipid metabolism, including fatty acid (FA) synthesis and cholesterol and steroid biogenesis; MPS2 has upregulated carbohydrate (citric acid/tricarboxylic acid (TCA) cycle and glycolysis) and purine and pyrimidine metabolism; and MPS3 is a mixed subtype of these pathways [12]. MPS1 consists of the LAR subtype, MPS2 is basal-like, and MPS3 consists of multiple molecular subtypes [12]. This metabolic heterogeneity of TNBC increases the difficulty of specific metabolic targeting [13].

Dietary phytochemicals are of keen interest for inhibiting BC metastatic disease because they show lower toxicity than synthetic drugs [14,15]. Anacardic acid (AnAc) is a natural, bioactive, phenolic lipid phytochemical that is routinely consumed in cashew apple juice [16] and mangos [17]. We reported that one purified AnAc congener (AnAc 24:1n-5, hereafter AnAc) inhibited proliferation and increased apoptosis in MCF-7 luminal A BC cells and MDA-MB-231 TNBC, but not primary human mammary epithelial cells (HMECs) [18].

We previously reported that RNA sequencing (seq) analysis of AnAc-treated MCF-7 and MDA-MB-231 BC cells revealed that AnAc reduced stearoyl-CoA desaturase 1 (*SCD*) and carnitine palmitoyltransferase 1A (*CPT1A*) mRNA abundance and increased endoplasmic reticulum stress (ERS) [19], mimicking the induction of ERS with *SCD* knockdown in lung cancer cells [20]. A limitation of transcriptomic data is the lack of concordance to protein and metabolic effects of cell treatments. Metabolomics provides an analysis of metabolites that are the end products of cellular biological processes in response to a specific treatment. To examine how AnAc affects metabolic programming in BC cells, we performed untargeted metabolomic analysis of luminal A MCF-7 and MDA-MB-231, MDA-MB-468, HCC1806, and BT-20 TNBC cells. In addition, we integrated the metabolomics identified in this study with previously established transcriptomic data that identified AnAc-regulated differentially expressed genes (DEGs) in MCF-7 and MDA-MBA-231 cells [19] to identify AnAc-targeted biological pathways in these cell lines. This is the first examination of the impact of AnAc on cellular metabolomes and the integration of AnAc-mediated changes in metabolomics and transcriptomics.

## 2. Results and Discussion

### 2.1. AnAc Inhibits TNBC Cell Viability

**Figure 1:**
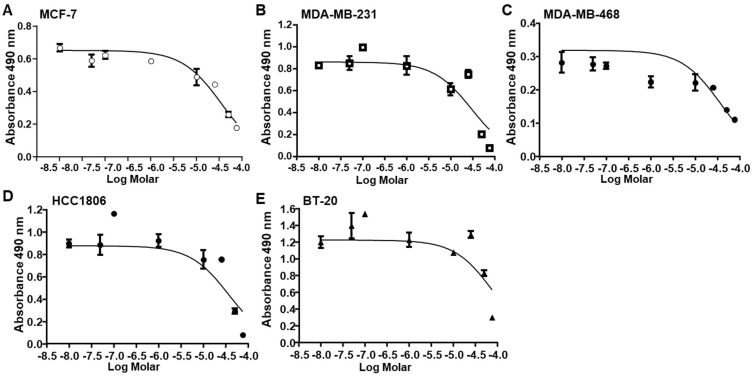
Anacardic acid (AnAc) inhibits the viability of ER+ and TNBC cells. Each cell line was grown in 5% DCC-FBS-phenol red-free IMEM for 48 h prior to 48 h treatment with EtOH (ethanol, the vehicle control) or the indicated concentration of AnAc for 48 h prior to MTT assay. Values are the mean + SEM of 4 replicates within one experiment. IC_50_ values from repeated experiments are in Table 2. (**A**–**E**) are the viability plots for MCF-7, MDA-MB-231, MDA-MB-468, HCC1806, and BT-20 BC cell lines, respectively.

As previously reported [18], the viability of MCF-7 luminal A BC cells and MDA-MB-231 TNBC cells was inhibited by AnAc with 48 h of treatment (Figure 1A,B). To determine if other TNBC cell lines are sensitive to AnAc, the viability of MDA-MB-468, HCC1806, and BT-20 cells (Table 1) was examined (Figure 1C–E). Parallel experiments were performed using a dsDNA quantification kit to quantify cellular DNA as an index of cell proliferation [21] (Supplementary Figure S1). The AnAc IC_50_ values for each cell line are summarized in Table 2.

### 2.2. Metabolome Changes in Response to AnAc in BC Cells

BC cell lines are accepted models for examining the impact of anti-cancer drugs because they recapitulate the heterogeneity of clinical samples [31]. To investigate the impact of AnAc on cellular metabolism, we treated MCF-7 luminal A BC cells and the four TNBC cell lines, namely MDA-MB-231, MDA-MB-468, HCC1806, and BT-20 (Table 1), with AnAc for 24 h (Table 2). Metabolites were identified by gas chromatography–mass spectrometry (GC/M)S-based metabolite profiling against standards as described in [32] and detailed in Section 4. A total of 193 metabolites were identified (Supplementary Table S1).

**Figure 2:**
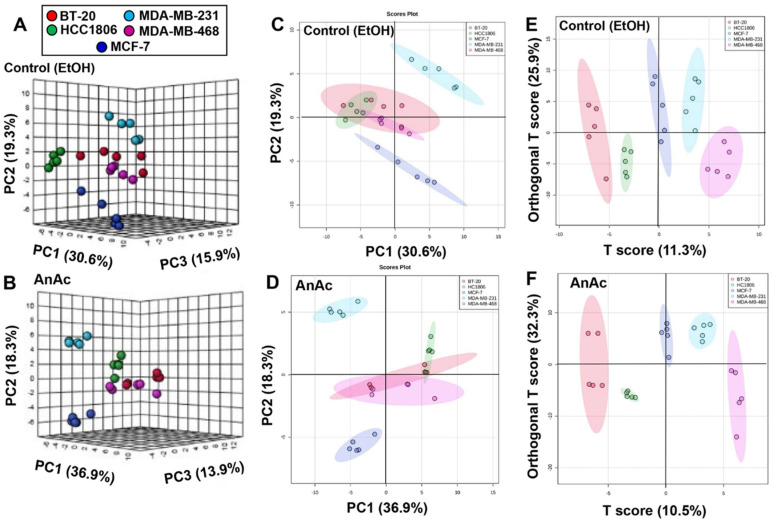
Principal Component Analysis (PCA) and Orthogonal Partial Least Squares - Discriminant Analysis (OPLS-DA) of metabolites from the control (EtOH) (**A**,**C**,**E**) and AnAc-treated (**B**,**D**,**F**) cell lines. Each point is a separate replicate sample from the indicated cell lines. Each cell line was grown in 5% DCC-FBS-phenol red-free IMEM for 48 h prior to 24 h treatment with EtOH (vehicle control) or AnAc for 24 h. PCA scores of plots of all metabolites: 3D score plots (**A**,**B**) and 2D score plots (**C**,**D**) of the same samples reveals a distinct metabolic signature of MCF-7 and MDA-MB-231 cells from the other three TNBC cell lines: BT-20, HCC1806 and MDA-MB-468. OPLSA-DA score plots identify distinct metabolic signatures of each cell line with BT-20 and HCC1806 showing lower T scores compared to MCF-7, MDA-MB-231 and MDA-MB-468 cells.

To determine whether the metabolomic signature of the cell lines differed between the control (EtOH) treatment and the AnAc treatment, we used MetaboAnalyst 6.0 software [33]. First, we performed unsupervised Principal Component Analysis (PCA) resulting in PCA score plots that visualized the effects of cell line and treatment on the cellular metabolome (Figure 2A–D). The 3D PCA score plots (Figure 2A,B) and 2D PCA score plots (Figure 2C,D) of the same samples reveal a distinct metabolic signature of MCF-7 (luminal A) and MDA-MB-231 (TNBC, basal B) cells from the other three TNBC cell lines: BT-20, HCC1806, and MDA-MB-468 (all basal A). PCA shows metabolic signatures for each cell line along the first principal component (PC1), explaining 30.6% (C) and 36.9% (D) of variance for the control and AnAc-treated cells, respectively. MCF-7 luminal A cells cluster in component 1 relative to the four TNBC cell lines in the second component with both control and AnAc treatment (Figure 2C,D). Taken together, the results of the PCA analysis reveal that the cell line has the most impact on the metabolic profiles. This is shown in a PCA analysis of all five cell lines +/− AnAc treatment in Supplementary Figure S2. Orthogonal Partial Least Squares-Discriminant Analysis (OPLS-DA), which better filtered out ‘noise’ within the five biological replicates in each cell line [34], revealed that each cell line had a distinctive metabolic cluster (Figure 2E,F). BT-20 and HCC1806 showed lower T scores in OPLS-DA compared to MCF-7, MDA-MB-231, and MDA-MB-468 cells (Figure 2E,F). OPLSA-DA score plots revealed distinct metabolic signatures with AnAc treatment of each cell line (Figure 2E,F).

**Figure 3:**
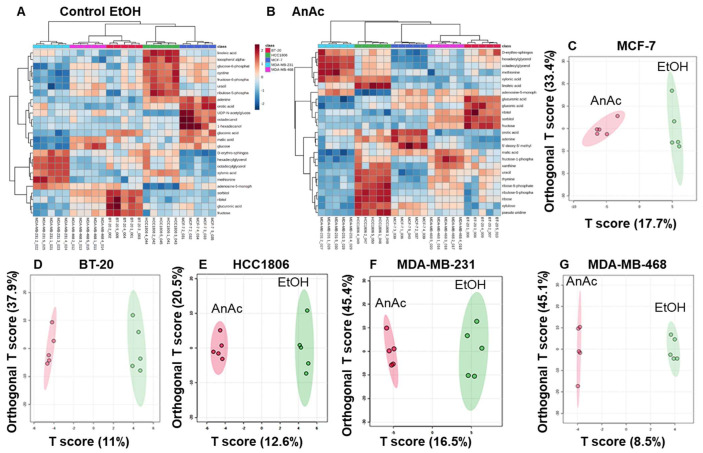
Metabolite differences between cell lines and in response to AnAc treatment. Heatmaps of unsupervised hierarchical cluster analysis of the top 25 metabolite concentrations in the metabolomics data from the control (EtOH) (**A**) and AnAc-treated (**B**) cell lines. The rows show different metabolites in EtOH (**A**) and AnAc-treated cells (**B**) with the columns indicating the individual cell lines. (**C**–**G**) OPLS-DA of metabolites from the control (EtOH) and AnAc-treated cell lines. (**C**): MCF-7, (**D**): BT-20, (**E**): HCC-1806, (**F**): MDA-MB-231, and (**G**): MDA-MB-468 BC cells. Each point is a separate replicate sample from the indicated cell lines. Each cell line was grown in 5% DCC-FBS-phenol red-free IMEM for 48 h prior to 24 h treatment with EtOH or AnAc. OPLSA-DA score plots identify distinct metabolic signatures with AnAc treatment of each cell line.

Heatmaps were generated to visualize differences in the 194 detected metabolites with control (EtOH) and AnAc treatment (Supplementary Figure S3) and the top 25 metabolites (Figure 3A,B). Unsupervised hierarchical clustering clearly separated the five BC cell lines. MDA-MB-468 and BT-20 were clustered in both EtOH- and AnAc-treated cells. HCC1806 clustered with MCF-7 only in EtOH-treated cells. In contrast, MDA-MB-231 cells did not pairwise cluster with any of the cell lines, perhaps reflecting their TNBC, basal B status (Table 1).

SAM (significance of metabolites) analysis identified 96 metabolites in EtOH-treated cells and 128 metabolites in AnAc-treated cells (Supplementary Tables S2 and S3). Sorbitol, hexadecylglycerol, octadecylglycerol, xanthine, ribulose-5-phosphate, and uracil were in common in the top 10 metabolites. Variable importance plots (VIPs) of the top 15 metabolites identified in OPLS-DA score plots show that 4-aminobutyric acid (GABA), glycine, and phytosphingosine contribute more to the EtOH-treated cell line separation (based on high concentrations in MDA-MB-468 and MDA-MB-231 TNBC cells, Supplementary Figure S4A). Enrichment analysis in MetaboAnalyst indicates these three metabolites are related to glutamate, alanine, and glutathione (GSH) metabolism and to carnitine synthesis, which agrees with previous metabolomics reports on MDA-MB-468 and MDA-MB-231 TNBC cells [35,36]. Guanine, fucose, cholesterone, and *trans*-4-hydroxyproline contribute more to the model in AnAc-treated cells (based on high concentrations in BT-20, MDA-MB-468, and HCC1806 TNBC cells and MCF-7 luminal A BC cells, respectively, Supplementary Figure S4B). Enrichment analysis identified fructose and mannose degradation, arginine and proline metabolism, and purine metabolism for these four metabolites. Similar to a previous metabolomic analysis of two TNBC cell lines, namely MDA-MB-231 and MDA-MB-468 [31], we observed metabolic diversity among the four TNBC cell lines, reflecting the heterogeneity of TNBC. Table 3 summarizes the AnAc-induced significant changes in metabolites in the five BC cell lines. The cell-line-specific changes in metabolites with AnAc treatment suggest differential alterations in multiple pools of cellular building blocks and nutrients affecting the inhibition of cell viability with AnAc treatment.

**Figure 4:**
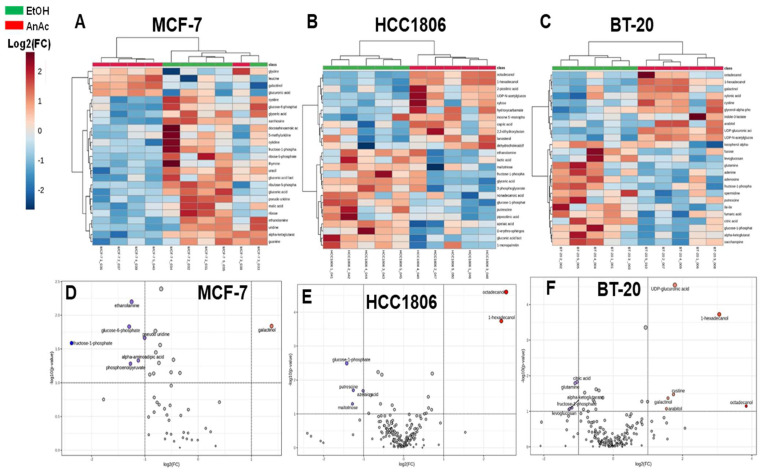
Analysis of AnAc-regulated metabolites in MCF-7, HCC1806 and BT-20 BC cells. Heatmaps of the top 25 identified metabolites from control (EtOH) and AnAc-treated MCF-7 (**A**), HCC1806 (**B**) and BT-20 (**C**) BC cells. Each column is an individual replicate. One AnAc-treated sample was sorted into the Control cluster. Volcano plots using a 2-fold cut off identified up and down-regulated metabolites in MCF-7 (**D**), HCC1806 (**E**) and BT-2 (**F**) BC cells. MetaboAnalyst identified selected metabolites (blue font). Red and purple dots indicate increased and decreased abundance, respectively.

OPLS-DA score plots revealed that each cell line had a distinctive metabolic cluster in response to AnAc treatment compared to EtOH control (Figure 3C–G). Thus, we analyzed the effect of AnAc on metabolites within each cell line. For MCF-7 cells, unsupervised hierarchical clustering generated a heatmap of the top 25 metabolites discriminating the cellular impact of AnAc (Figure 4A). One AnAc-treated sample clustered with the EtOH control-treated samples (Figure 4A). Volcano plot analysis using a 2-fold cut-off (Figure 4D) revealed that only one metabolite, galactinol, was significantly increased in response to AnAc, whereas 16 metabolites were reduced (Table 3). Galactinol is a product of galactose metabolism and was also increased in AnAc-treated MDA-MB-468 TNBC cells. Among the decreased metabolites, ribulose 5-phosphate and ribose 5-phosphate suggest a decrease in the pentose phosphate pathway (PPP). Gluconic acid, a metabolite of glucose, was reduced with AnAc treatment of MCF-7 cells and is increased in the plasma and urine of BC patients (reviewed in [37]). In contrast, gluconic acid was increased in AnAc-treated MDA-MB-468 TNBC cells, implicating differences in glucose metabolism in these two BC cell lines. VIPs of the top 15 metabolites identified as increased or decreased by AnAc treatment of MCF-7 cells are shown in Supplementary Figure S5A,B. Enrichment analysis in MetaboAnalyst implicates AnAc-mediated decreases in galactose and pyrimidine metabolism, glycolysis, gluconeogenesis, and lactose and trehalose degradation (Supplementary Figure S5C).

For HCC1806 and BT-20 TNBC cells, unsupervised hierarchical clustering generated a heatmap clearly separating the AnAc-treated samples from the EtOH control-treated samples (Figure 4B,C). Volcano plot analysis using a 2-fold cut-off (Figure 4E,F) revealed significant increases in octadeconol and 1-hexadecanol with AnAc treatment in HCC1806 cells, whereas four metabolites were significantly reduced: glucose-1-phosphate, putrescene, azelaic acid (1,7-heptanedicarboxylic acid), and maltotriose (Table 3). Octadecanol is stearyl alcohol, a metabolite of stearic acid. 1-hexadecanol, a product in the FA degradation pathway, is a volatile organic compound that is a potential biomarker for cancer and is found in breast cancer cells [38]. Plasmalogen synthesis, galactose metabolism, and spermidine and spermine synthesis were the most significant pathways in enrichment analysis (Supplementary Figure S5F). Plasmalogens refer to lipids classified as phosphatidylethanolamines (PEs) by the fatty acyl linkage to the glycerol backbone and include vinyl ether-linked fatty acyls in the sn-1 position (1-alkenyl-2-acyl) [39]. Plasmalogens are considered to protect lipids from free radical damage.

VIPs of the top 15 metabolites increased or decreased by AnAc treatment of BT-20 cells are shown in Supplementary Figure S6A,B. Interestingly, 1-hexadecanol was also increased in AnAc-treated BT-20 cells, as seen in HCC1806 and MDA-MB-468 cells (Table 3), and all three basal A TNBC cell lines (Table 2). Enrichment analysis in MetaboAnalyst suggested an increase in the Warburg Effect (due to the decrease in citric acid and glutamine), decreased phenylacetate and increased nucleotide sugar metabolism, and decreased transfer of acetyl groups into mitochondria, thus reducing the TCA cycle, as significantly associated with these six AnAc-mediated changes in BT-20 metabolites (Supplementary Figure S7A). AnAc increased cystine (DL-cystine) in BT-20 cells but decreased cystine in MCF-7 cells (Table 3). Cystine is a metabolite from the oxidation of cysteine that contains two cysteine molecules linked by a disulfide bond. TNBC cells are “cysteine-addicted”, and cysteine deprivation triggered programmed necrosis in BT-20, MDA-MB-231, and MDA-MB-157 TNBC cell lines [40]. Continuous cysteine import is required for de novo GSH synthesis to protect basal TNBC cells from higher levels of oxidative stress [40].

**Figure 5:**
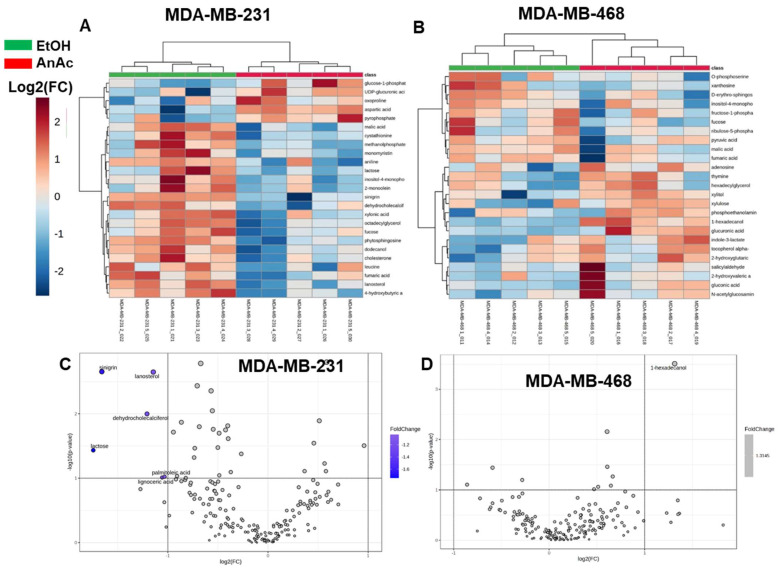
Analysis of AnAc-regulated metabolites in MDA-MB-231 and MDA-MB-468 TNBC cells. Heatmaps of the top 25 identified metabolites from control (EtOH) and AnAc-treated MDA-MB-231 (**A**) and MDA-MB-468 (**B**) TNBC cells. Each column is an individual replicate. One AnAc-treated sample was sorted into the Control cluster. Volcano plots using a 2-fold cut off identified up and down-regulated metabolites in MDA-MB-231 (**C**) and MDA-MB-468 (**D**) TNBC cells. MetaboAnalyst identified selected significant metabolites (blue font). Red and purple dots indicate increased and decreased abundance, respectively.

Similarly, heatmaps separating metabolic effects of control (EtOH) and AnAc treatment of MDA-MB-231 and MDA-MB-468 TNBC cells and Volcano plot analyses were performed (Figure 5A–D). VIPs of the top 15 metabolites identified as increased or decreased by AnAc treatment of MDA-MB-231 and MDA-MB-468 TNBC cells plotted by OPLS-DA scores are shown in Supplementary Figure S6C–F. AnAc treatment of MDA-MB-231 cells increased aspartic acid and pyrophosphate and decreased ten metabolites (Table 3). Enrichment analysis in MetaboAnalyst (excluding octadecylglycerol which was not included in this tool) indicated lactose synthesis, urea cycle, inositol metabolism, ammonia recycling, aspartate and galactose metabolism, steroid biosynthesis, and glutamate metabolism as the top enrichment pathways with AnAc treatment of MDA-MB-231 cells (Supplementary Figure S7B). 1-hexadecanol was also increased with AnAc treatment of MDA-MB-468 cells, as detected in HCC1806 and BT-20 TNBC cells (Table 3). Enrichment analysis in MetaboAnalyst indicated plasmalogen synthesis as the top enrichment pathway based on the changes in these four metabolites with AnAc treatment of MDA-MB-468 cells (Supplementary Figure S7C).

### 2.3. Pathway Analysis of the Effect of AnAc on Metabolites in BC Cell Lines

MetaCore pathway enrichment analysis was used to identify cellular pathways based on the input of the metabolites altered by AnAc in the cell lines. For this unbiased analysis, all AnAc-regulated metabolites in each cell line were included in the analysis. The top 10 pathways for MCF-7, BT-20, HCC1806, MDA-MB-231, and MDA-MB-468 cells are shown in Supplementary Tables S4A–S8A. A Venn diagram of the top ten pathways for AnAc-regulated metabolites identified for each cell line shows one common pathway for three cell lines: Signal transduction Amino acid-dependent mTORC1 activation in MCF-7, BT-20, and MDA-MB-231 cells (Supplementary Figure S8). MCF-7 and BT-20 cells had two additional pathways in common: Metabolism in pancreatic cancer cells; Immune response: Distinct metabolic pathways in naive and effector CD8+ T cells (Supplementary Figure S8). HCC1806 and BT-20 cell lines showed two pathways in common: Mechanisms of drug resistance in multiple myeloma; Influence of bone marrow cell environment on progression of multiple myeloma. There were no overlapping pathways identified for MDA-MB-231 cells, reflecting the intrinsic metabolic differences in this cell line from the other TNBC cell lines and MCF-7 cells examined in this study as seen in heatmaps (Supplementary Figure S3). We then compared the pathways identified using the significant metabolites identified by Volcano plot analysis in MetaboAnalyst with those generated by MetaCore’s analysis of the AnAc-regulated metabolites in each cell line (Supplementary Tables S4B–S8B). As anticipated, given the common input metabolites, a high degree of similarity was apparent.

**Figure 6:**
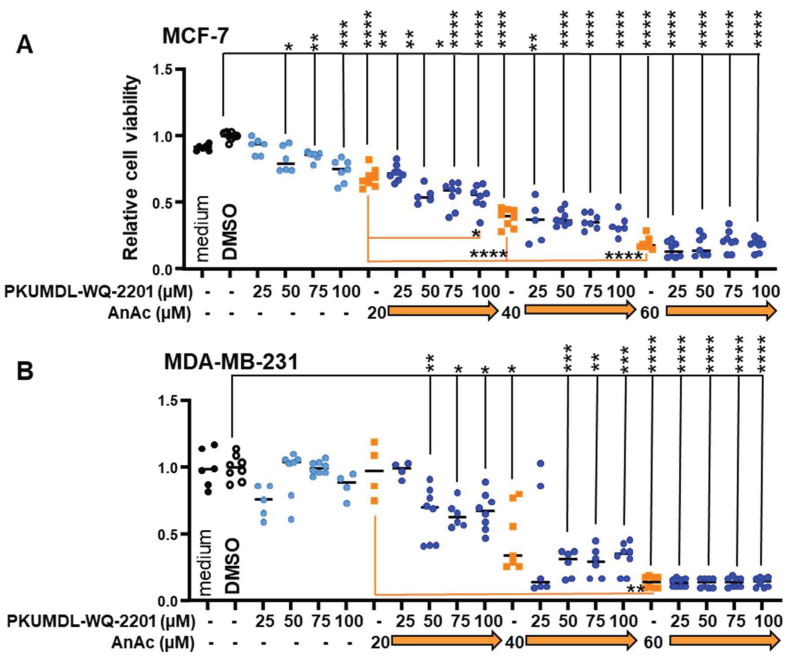
AnAc and PDGH inhibitor PKUMDL-WQ-2201 reduce MCF-7 and MDA-MB-231 cell viability. MCF-7 (**A**) and MDA-MB-231 (**B**) cells were treated with vehicle control (DMSO), PHGDH inhibitor PKUMDL-WQ-2201 or AnAc at the indicated concentrations for 48 h. MTT assays were performed and data were analyzed by one-way ANOVA followed by Tukey’s multiple comparison test. Individual values from two biological replicate experiments were plotted. * *p* < 0.05, ** *p* < 0.01, *** *p* < 0.001, **** *p* < 0.0001.

Supplementary Table S4A indicates that “Glycine and L-serine metabolism” was the second enriched pathway identified in AnAc-treated MCF-7 cells, and reports have demonstrated that the growth of TNBC cells, including MDA-MB-231, is inhibited by phosphoglycerate dehydrogenase (PHDGH) inhibition [41]. PHGDH is the first and one of the rate-limiting steps in the serine synthesis pathway (SSP) that is upregulated in breast and other metastatic cancers [42]. We previously reported that the PHGDH inhibitor PKUMDL-WQ-2201 sensitized tamoxifen-resistant LCC9 cells to 4-hydroxytamoxifen [43]. To determine if AnAc would sensitize BC cells to PKUMDL-WQ-2201 inhibition, MTT assays were performed. PKUMDL-WQ-2201 inhibited the viability of MCF-7, but not MDA-MB-231 cells (Figure 6). Similar results were reported for these non-PHGDH-amplified BC cell lines [44]. The combination of 20 μM AnAc **+** 100 μM KUMDL-WQ-2201 showed an additive effect in inhibiting MCF-7 viability. Future experiments addressing combinations of AnAc with other metabolic pathway inhibitors will be important to determine if AnAc will sensitize BC cells to metabolic inhibitors.

The top two pathways ‘driven’ by the most significant metabolite changes identified for each cell line with AnAc treatment are shown in Supplementary Figures S9–S13. “Signal transduction: amino acid-dependent mTORC1 activation” was the top pathway identified by metabolite expression in AnAc-treated MCF-7 (Supplementary Table S4A) and MDA-MB-231 cells (Supplementary Table S7A) and the third pathway in AnAc-treated BT-20 cells (Supplementary Table S6A). mTORC1 activity is dynamically regulated to respond to various upstream signaling factors including amino acids, e.g., the increase in leucine in these three BC cell lines with AnAc treatment, energy, and growth factors, and is a target of BC therapy. Everolimus, an mTOR inhibitor, is FDA-approved for use in combination with aromatase inhibitor (AI) therapies for metastatic BC in ER+/HER2 postmenopausal (PM) women after AI treatment failure with letrozole or anastrozole [45], but it was not effective in TNBC patients in phase II clinical trials [46]. BC prevention with Everolimus in high-risk women remains of interest [47]. However, our analysis suggests that BC patients with metastatic ER+ disease on Everolimus might benefit from AnAc or that TNBC patients might benefit from the combination of Everolimus and AnAc, whether in food or beverage sources or as a supplement, which will require a clinical trial.

The pathway map “Immune response: The effect of IDO1 on T cell metabolism” was identified by the inclusion of different metabolites in AnAc-treated luminal A MCF-7 BC cells (decreased alpha-ketoglutarate, phosphoenolpyruvate, D-ribose 5-phosphate) versus BT-20 TNBC cells (decreased citric acid and L-glutamine) (Supplementary Tables S4A and S6A). TNBC cells are ‘glutamine addicted’ [48]. The decreases in lanosterol, dehydrocholecalciferol, and lactose in AnAc-treated MDA-MB-231 cells (Table 3) were associated with decreased cholesterol biosynthesis, vitamin D_3_ metabolism, and galactose metabolism in MetaCore Pathway Maps analysis. The pathway “L-arginine metabolism” was identified in both HCC1806 and MDA-MB-231 TNBC cells (Supplementary Tables S5A and S7A). However, putrescine (decreased) was the only metabolite matching “L-arginine metabolism” in HCC1806 cells, whereas putrescine was not included in the AnAc-regulated metabolites in MDA-MB-231 cells. Instead, increased aspartic and fumaric acids matched the “L-arginine metabolism” in AnAc-treated MDA-MB-231 cells. Arginine is a precursor for putrescine and polyamine biosynthesis. TNBC is dependent on the uptake of extracellular arginine [49]. The pathways “aminoactyl-tRNA biosynthesis in mitochondrion” and “in cytoplasm” were identified in MDA-MB-231 cells (Supplementary Table S7A). For AnAc-treated MDA-MB-468, the decrease in sphingosine was featured in all the pathways in MetaCore analysis and 8 of the 10 pathways using the VIP analysis in MetaboAnalyst (Supplementary Table S8A,B). Ceremide and its sphingolipid metabolites are key regulators of cancer cell signaling including proliferation, resistance to chemotherapy and immunotherapy, and cellular stress responses [50]. The potential of targeting sphingosine-1-phosphate (S1P) to inhibit BC metastatic spread is of keen interest with a number of FDA-approved and pre-clinical drugs targeting S1P [51] that could potentially be examined in combination with AnAc in TNBC. Taken together, these data reflect the diversity of metabolic responses to AnAc detected in the five BC cell lines seen in PCA (Figure 2D,F) and heatmap (Figure 3B) analyses.

We then compared the pathways identified using the significant metabolites identified by Volcano plot analysis in MetaboAnalyst with those generated by MetaCore’s analysis of the AnAc-regulated metabolites in each cell line. Some of the pathways identified were identical, but the order of the pathways and some of the metabolites included were different between the analyses of the same cell line. For example, for MCF-7 cells using the significant AnAc-regulated metabolites identified by MetaboAnalyst, the top pathway was “Immune response: The effect of IDO1 on T cell metabolism” (Supplementary Figure S9C), which was the fourth pathway identified in the MetaCore analysis (Supplementary Table S4B), and different metabolites were included in each analysis (Supplementary Table S4A versus Supplementary Table S4B). One difference is that the MetaCore analysis on the AnAc-regulated metabolites in MCF-7 cells appears from the output to focus more on the AnAc-upregulated metabolites, whereas after metabolites were filtered in MetaboAnalyst and the Volcano plot was generated, more AnAc downregulated metabolites are seen compared to one significant upregulated metabolite (galactinol) in MetaboAnalyst (Figure 4B). The MetaCore pathway analysis identified downregulated metabolites in pathways suggesting reduced glycolysis, decreased pentose phosphate pathway and nucleotide biosynthesis, and decreased FA biosynthesis in AnAc-treated MCF-7 cells (Supplementary Figure S9B). Inhibition of these pathways is commensurate with the observed AnAc-mediated inhibition of MCF-7 cell viability (Figure 1).

For HCC1806 TNBC cells, glycogen metabolism was the top pathway in both MetaCore and MetaboAnalyst analyses (Supplementary Table S5A,B, Supplementary Figure S10A,C). Inhibitors of glycogen synthase kinase-3 (GSK-3) have been suggested to be a therapeutic target in BC [52] and could be tested in combination with dietary AnAc in future clinical trials. A limitation is that AnAc is the main allergen of cashew allergy (reviewed in [53]). However, studies in Balbc mice showed that the acute minimum lethal dose of >2 g/kg, suggesting AnAc at the concentrations used here may be tested in mouse BC models [54]. All six pathways and metabolites in the MetaboAnalyst were identical to those identified in the MetaCore analysis, but MetaCore included UDP-N-acetyl-D-glucosamine (UDP-N-acetylglucosamine, UDP-GlcNAc, which was increased in AnAc-treated HCC1806 cells) in four additional pathways not seen in MetaboAnalyst analysis. UDP-GlcNAc is a product of the hexosamine biosynthesis pathway that contributes to O-linked β-GlcNAc modifications on intracellular proteins that are elevated in cancer cells linked to the activation of the AKT and mTOR pathways [55]. UDP-GlcNAc was lower in MDA-MB-231 and MDA-MB-468 TNBC cells than in luminal A MCF-7 and BT474 BC cells [31].

“Immune response: Distinct metabolic pathways in naive and effector CD8+ T cells” was the top pathway for AnAc-treated BT-20 TNBC cells in both MetaCore and MetaboAnalyst analyses, but included alpha-ketoglutarate which was reduced with AnAc treatment (Supplementary Table S6A,B, Supplementary Figure S11A,C). Two pathways, “urea cycle” and “de novo IMP biosynthesis”, were identified by metabolites in AnAc-treated MDA-MB-231 cells by both MetaCore and MetaboAnalyst, but MetaCore included fumaric acid (the conjugate acid of fumarate in the TCA cycle) in addition to aspartic acid in these pathways (Supplementary Table S7A,B). As indicated above, for AnAc-treated MDA-MB-231 TNBC cells, “Signal transduction_Amino acid-dependent mTORC1 activation” was the top pathway identified by MetaCore analysis, whereas “Neurophysiological process_Role of CDK5 in presynaptic signaling” and “Prolactin signaling in prostate cancer” were the top two pathways from the MetaboAnalyst analysis (Supplementary Figure S12A–D).

“Cholesterol and Sphingolipid transport” and “Cholesterol and Sphingolipid transport/Distribution to the intracellular membrane compartments” were the top two pathways in AnAc-treated MDA-MB-468 cells in MetaCore analysis (Supplementary Figure S13A,B). MetaboAnalyst identified “Signal transduction_Role of Sphingosine 1-phosphate as an intracellular mediator” and “Apoptosis and survival_Ubiquitination and phosphorylation in TNF-alpha-induced NF-kB signaling” with these changes in metabolites in AnAc-treated MDA-MB-468 TNBC cells (Supplementary Figure S13C,D). We reported that AnAc inhibits TNF-induced NF-kB activity in MCF-7 cells [19].

### 2.4. Integration of Multi-Omics Analysis of the Effect of AnAc in MCF-7 and MDA-MB-231 Cells

We previously performed mRNA and miRNA-seq analysis in MCF-7 and MDA-MB-231 cells treated with 13.5 and 35 µM AnAc, respectively, for 6 h [19,56]. GO (Gene Ontology) processes in AnAc-treated MCF-7 and MDA-MB-231 cells included “Triglyceride metabolic processes” for downregulated DEGs and “Acetyl CoA biosynthetic process from pyruvate” for upregulated DEGs [19]. In our analysis of upregulated miRNAs in AnAc-treated MCF-7 and MDA-MB-231 cells, common GO processes included “cellular response to inorganic substance” [56]. There were no common miRNAs reduced by AnAc treatment in both MCF-7 and MDA-MB-231 cells [56]. For miRNAs uniquely downregulated in MCF-7 in response to AnAc, the second GO process was “Unsaturated FA biosynthesis”, and for miRNAs downregulated in AnAc-treated MDA-MB-231 cells, the second GO process was “Apoptosis and survival: Endoplasmic reticulum stress response pathway” [56].

MetaCore was used to integrate the DEG (mRNA-seq) transcriptome and metabolome data of MCF-7 and MDA-MB-231 cells in response to AnAc. We acknowledge the limitation that MCF-7 and MDA-MB-231 cells were treated for 24 h and with 28 and 20 μM AnAc, respectively, for metabolome analysis reported here, i.e., longer times and different AnAc concentrations than the RNA-seq analysis. However, the 6 h time point for RNA-seq analysis will primarily reflect early gene changes in response to AnAc, while the 24 h exposure prior to metabolomics analysis allows accumulation of metabolic changes driven by AnAc treatment that are the final products of various biological processes upstream that are expected to be captured in DEGs in mRNA-seq. Future studies examining intermediate and later time points are needed to construct a timeline of AnAc-mediated molecular, metabolic, and proteome changes in BC cells.

Integration of the mRNA-seq DEG data and metabolome data from AnAc-treated MCF-7 revealed two pathways (Table 4, Supplementary Figures S13B and S14A): “Regulation of lipid metabolism: Fatty acid-dependent regulation of lipidic metabolism”, which is indicated as statistically significant, and “Signal transduction: mTORC1 downstream signaling”, which was not statistically significant. In the “Regulation of lipid metabolism” pathway, the increase in linoleic acid with AnAc treatment was linked in MetaCore pathway analysis with increased transcript abundance of PDK4 (pyruvate dehydrogenase kinase-4) which is transcriptionally upregulated by linoleic acid-activated PPARα [57] as depicted in Supplementary Figure S14A. PDK4 is increased by glucocorticoids [58] and inhibited by insulin in BC cells [59]. PDK4 phosphorylates and inactivates pyruvate dehydrogenase (PDH) which would reduce acetyl CoA for FA biosynthesis and conversion to citrate for the TCA cycle. The reduction in the INSIG transcript would reduce its abundance in the endoplasmic reticulum membrane where it binds SCAP to promote SREBP’s endoplasmic reticulum retention, hence blocking SREBP1 activation and inhibiting FA synthesis. The decrease in ribose-5-phosphate by AnAc suggests inhibition of the PPP (Supplementary Figure S14B), while the decrease in SCD is connected to reduced FA and lipid biosynthesis and inhibition of cell growth, as seen in Figure 1, and to alpha linolenic and linoleic acids (Supplementary Figure S5C). SCD (also called SCD-1) converts the lipid stearate into oleate (oleic acid) and palmitate into palmitoleate (palmitoleic acid). Oleic acid and palmitoleic acid are monounsaturated FAs (MUFAs) that are incorporated into triglycerides (TGs) and cholesterol esters (CEs) for plasma membrane fluidity. Patients whose primary breast tumors express high SCD levels have shorter RFS and OS [60]. We detected higher SCD protein abundance in TNBC cell lines relative to MCF-7 luminal A BC cells (Supplementary Figure S15). SCD is a target of clinical interest for treating breast and other cancers [61]. Here, we did not detect a significant reduction in either oleic and palmitoleic acids in AnAc-treated MCF-7 BC cells or any of the TNBC cell lines.

Our previous enrichment analysis of DEGs in AnAc-treated MCF-7 and MDA-MB-231 BC cells revealed increased endoplasmic reticulum stress (ERS) [19]. Of the metabolites detected here, none were linked with ERS in enrichment pathway analysis; however, the decrease in DHA in MCF-7 cells and the decrease in dehydrocholecalciferol and lanosterol in MDA-MB-231 cells (Table 3) suggest ERS [62] and impaired cholesterol/steroid biosynthesis at the ER membrane [63]. Targeted lipidomics metabolite analysis of AnAc-treated BC cells will be needed to further examine lipid changes associated with ERS.

For MDA-MB-231 cells, 27 pathways were identified in the integration of metabolomics and mRNA-seq data, with 17 showing a minimum *p* value < 0.05 (Table 5). This analysis revealed pathways driven by the more numerous changes in the mRNA transcriptome than alterations in the metabolome of AnAc-treated MDA-MB-231 cells. The top pathway associated with the transcriptome and metabolome for AnAc-treated MDA-MB-231 cells was “Signal transduction: Amino acid-dependent mTORC1 activation” with increased leucine in the metabolome and increased SESN1 (Sestrin 1), SLC3A2 (Solute Carrier Family 3 Member 2), SLC1A5 (Solute Carrier Family 1 Member 5, ASCT2), IPMK (inositol polyphosphate multikinase), and SLC38A2 (Solute Carrier Family 38 Member 2) in the RNA-seq data (Table 5, Supplementary Figure S16A). Increased plasma SESN1 was associated with short RFS in TNBC patients [64]. SLC3A2 is an amino acid transporter and also modulates integrin-induced signal transduction, which drives malignant tumor cell spreading and migration associated with decreased OS in BC patients [65]. SLC1A5 (ASCT2) controls glutamine uptake, which is critical for TNBC cell viability, and is transcriptionally upregulated by MYC [66], which was upregulated in AnAc-treated MDA-MB-231 cells [19], also seen in the fourth pathway in Table 5, Supplementary Figure S16D. SLC38A2 is another glutamine transporter overexpressed in TNBC and associated with lower OS [67]. IPMK is involved in many functional protein–protein interactions beyond phospholipid metabolism to maintain cell membrane integrity, including as a transcriptional regulator of arginine metabolism, a cofactor in mTOR signaling, and a regulator of AMPK [68]. IMPK enhances YAP/TEAD complex formation to increase YAP/TAZ targets promoting BC motility [69].

The next pathway was “Mechanisms of drug resistance in multiple myeloma”, which includes an increase in UDP-D-glucuronic acid; increased IL-6, IL-8, FZD2 (Frizzled), TCF3, and MYC; and a reduction in HRAS, HES1, and CCND1 (Cyclin D1) (Supplementary Figure S16B). The decrease in CCND1 is commensurate with AnAc inhibition of MDA-MB-231 cell viability (Figure 1). The third and fourth pathways were “Urea Cycle”, based on increased fumaric acid, aspartic acid, and Aquaporin 3, and “De novo IMP biosynthesis”, also featuring increased fumaric and aspartic acids (Table 5 and Supplementary Figure S16B–D). These data confirm BC cell-line-specific responses to AnAc at the transcriptome and metabolome levels and reflect concordances with pathways associated with metabolic and transcriptional changes induced by AnAc treatment of BC cells.

## 3. Conclusions

Here, we demonstrated that AnAc inhibited the viability of three additional TNBC cell lines, MDA-MB-468, HCC1806, and BT-20, in addition to our previous report that AnAc inhibited the cell proliferation of MDA-MB-231 TNBC and MCF-7 luminal A BC cells, but not primary human mammary epithelial cells in vitro [18]. We identified distinct basal (control) and AnAc-mediated metabolic signatures in each of the five BC cell lines, indicating that the cell line has the most impact on the metabolic profiles. We also observed a distinct metabolic signature of MCF-7 (luminal A) and MDA-MB-231 (TNBC, basal B) cells from the other three TNBC cell lines: BT-20, HCC1806, and MDA-MB-468 (all basal A). MetaboAnalyst and MetaCore enrichment analyses identified little overlap in pathway responses to AnAc in these cells. To our knowledge, this is the first integration of transcriptomic and metabolomic data from AnAc-treated cancer cells. Further investigations will be required to determine the time course of direct targets of AnAc-mediated alterations in metabolism and the mechanisms by which AnAc elicits these events in BC cells. Understanding the networks of metabolism changes with AnAc treatment will help unravel critical targets for AnAc’s anti-cancer activity in luminal A and TNBC cells.

### Limitations

Despite the novelty of the findings reported here, there are a number of limitations to our study. First, we included only one luminal A breast cancer cell line, MCF-7, in comparing control and AnAc treatment with four TNBC cell lines in vitro. Others reported distinct differences in metabolites of ER+ (T47D, MCF-7, HCC1428, ZR-75-1) versus TNBC (MDA-MB-231, MDA-MB-468, HCC70, and HCC1806) growth as tumor xenografts in vivo [70]. Second, for integrating transcriptomic and metabolomic data from MCF-7 and MDA-MB-231 cells, the cells were treated with 13.5 and 35 μM AnAc, respectively, for 6 h prior to RNA isolation and processing for Illumina RNA-seq [19], whereas for metabolome analysis, the cells were treated for 48 h with 28 μM (MCF-7) and 20 μM (MDA-MB-231) AnAc. Thus, it is likely that ~33% of the cells that underwent apoptosis and floated off the plates were excluded from this metabolome analysis.

## 4. Materials and Methods

### 4.1. Materials

AnAc 24:1n5 was purified to greater than 95% purity as previously reported [18,71]. AnAc 24:1n5 was dissolved in ethanol (EtOH); thus, EtOH was used as a vehicle control. PKUMDL-WQ-2201 was purchased from Sigma-Aldrich (cat. # SML1965, St. Louis, MO, USA) and was dissolved in 100% dimethyl sulfoxide (DMSO).

### 4.2. Cell Culture and Treatments

MCF-7, MDA-MB-231, MDA-MB-468, HCC1806, and BT-20 breast cancer cells were purchased from American Type Tissue Collection (ATCC, Manassas, VA, USA). All cell lines were verified by short tandem repeat (STR) genotyping (Genetica, LabCorp, Burlington, NC, USA). STR profiles were compared with publicly available profiles using Cellosaurus STR (ExPASy). MCF-7 cells were maintained in IMEM (Cellgro, Manassas, VA, USA) containing 10% fetal bovine serum (FBS, Atlanta Biologicals, Lawrenceville, GA, USA) and 1% Penicillin/Streptomycin (Cellgro). Table 1 lists the properties of the TNBC cell lines and their growth medium. For metabolomics, cells were grown to 75–80% confluence in phenol red-free IMEM (ThermoFisher, Waltham, MA, USA) medium containing 5% dextran-coated charcoal (DCC)-stripped FBS (hormone-depleted medium) in a T-75 flask for 48 h prior to treatment with the ~IC_50_ for AnAc 24:1n5 (hereafter AnAc) for 24 h (Table 2). Five independent replicate experiments were performed. The cells were pelleted by centrifugation at 2000 rpm (671× *g*) for 2 min, and the pellet was washed twice with ice-cold PBS and re-pelleted in a cryo tube. Cell pellets were flash frozen in liquid N_2_ and stored in liquid N_2_ prior to shipping to West Coast Metabolomics Center (https://metabolomics.ucdavis.edu/, accessed on 12 April 2024) for analysis. Supplementary Figure S17 presents a flow chart model of the experimental design.

### 4.3. Metabolomics

Metabolites were identified by GC/MS-based metabolite profiling against standards as described in [32]. Raw data files were preprocessed directly after data acquisition and stored as ChromaTOF-specific *.peg files, as generic *.txt result files, and additionally as generic ANDI MS *.cdf files. ChromaTOF vs. 2.32 was used for data preprocessing without smoothing, with a 3 s peak width, baseline subtraction just above the noise level, and automatic mass spectral deconvolution and peak detection at signal/noise levels of 5:1 throughout the chromatogram. Apex masses were reported for use in the BinBase algorithm. Result *.txt files were exported to a data server with absolute spectra intensities and further processed by a filtering algorithm implemented in the metabolomics BinBase database.

The BinBase algorithm (rtx5) used the following settings: validity of chromatogram (<10 peaks with intensity > 10^7^ counts s^−1^), unbiased retention index marker detection (MS similarity > 800, validity of intensity range for high *m*/*z* marker ions), retention index calculation by 5th order polynomial regression. Spectra were cut to 5% base peak abundance and matched to database entries from most to least abundant spectra using the following matching filters: retention index window ±2000 units (equivalent to about ±2 s retention time), validation of unique ions and apex masses (unique ion must be included in apexing masses and present at >3% of base peak abundance), mass spectrum similarity must fit criteria dependent on peak purity and signal/noise ratios and a final isomer filter. Failed spectra were automatically entered as new database entries if s/n > 25, purity < 1.0, and presence in the biological study design class was >80%. All thresholds reflected settings for ChromaTOF v. 2.32. Quantification was reported as peak height using the unique ion as default, unless a different quantification ion was manually set in the BinBase administration software BinView (rtx5). A quantification report table was produced for all database entries that were positively detected in more than 10% of the samples of a study design class (as defined in the miniX database) for unidentified metabolites. A subsequent post-processing module was employed to automatically replace missing values from the *.cdf files. Replaced values were labeled as ‘low confidence’ by color coding, and for each metabolite, the number of high-confidence peak detections was recorded, along with the ratio of the average height of replaced values to high-confidence peak detections. These ratios and numbers were used for the manual curation of automatic report data sets. The unique chemical identifier defined by the IUPAC and NIST consortia was included. The ‘KEGG’ identifier gave the unique identifier associated with an identified metabolite in the community database KEGG LIGAND DB. The ‘PubChem’ column denoted the unique identifier of a metabolite in the PubChem database. The actual data were peak heights for the quantification ion (m/z value) at the specific retention index. Heights were provided by West Coast Metabolomics instead of peak areas because peak heights are more precise for low-abundance metabolites than peak areas, due to the larger influence of baseline determinations on areas compared to peak heights. The complete data from West Coast Metabolomics are in Supplementary Table S1.

### 4.4. In Silico Analysis

MetaboAnalyst 6.0 was used for data analysis and interpretation [33]. Interquartile Data Range (IQR) was used to filter out metabolites that remained unchanged in all samples. Data were normalized using a generalized log transformation and Pareto scaling. PCA and OPLS-DA were performed. VIP scores and plots for the top 15 metabolites discriminating between cell lines or treatment groups were calculated in the OPLS-DA analysis. Heatmaps were created to visually identify metabolites with distinct high/low intensities, and the top 25 metabolites were ranked by *t*-test/ANOVA within the MetaboAnalyst 5.0 software. Both chemometrics analysis and hierarchical clustering analysis were performed.

MetaCore version 21.1 (Gene Go, Clarivate, Philadelphia, PA, USA) was used for independent metabolome analysis and integration of the metabolomics data with the RNA-seq transcriptome data for MCF-7 and MDA-MB-231 cells [19], pathway, and network analysis. Mapping functions within MetaCore involved calculating the statistical relevance of the matches found including public ontologies such as Gene Ontology (GO, www.geneontology.org, accessed within MetaCore on 12 April 2024). Ontologies were represented by canonical Pathway Maps, Process Networks, and GO Processes.

### 4.5. Integrative Analysis of Metabolomics and Transcriptomics

Differential metabolites (DMs) in AnAc-treated MCF-7 and MDA-MB-231 cells (VIP > 1.7, *p* < 0.05) and the respective DEGs from the RNA-seq analysis of AnAc-treated MCF-7 and MDA-MB-231 cells, data GSE78011 in the GEO database [19], were used for integrative analysis of the metabolic and transcription groups in MetaCore using Pathway Maps analysis.

### 4.6. Venn Diagram

The Venn diagram analysis for the top ten Pathway Maps identified for DMs in each of the five BC cell lines used (http://bioinformatics.psb.ugent.be/webtools/Venn,accessed on 20 May 2024).

### 4.7. Western Blots

Whole-cell extracts (WCEs) were prepared from MCF-7 and MDA-MB-231 cells as previously described [72]. Protein concentrations were determined (BioRad DC protein assay, Bio-Rad Laboratories, Hercules, CA, USA), and 25 µg of protein was separated on 10% SDS-PAGE gels and transferred to PVDF membranes that were blocked as previously described [73]. Membranes were incubated with a primary antibody against SCD (SCD1) #23393-1-AP1 (Protein Tech, Rosemont, IL, USA) and washed with TBS-Tween, followed by incubation with anti-rabbit (#7074S) (Cell Signaling Technology, Danvers, MA, USA) secondary antibodies. Membranes were incubated with Clarity Western ECL (Bio-Rad) and imaged on a Bio-Rad ChemiDoc™ XRS+ System with Image Lab™ Software (Bio-Rad). Blots were stained with Ponceau S for protein normalization.

### 4.8. Cell Titer MTT Assay and FluoReporter™ Blue Fluorometric dsDNA (Double-Stranded DNA) Quantification Assay

Cells were plated at 3000 cells per well of a 96-well plate for both MTT and dsDNA Quant assays in growth media and allowed to adhere overnight, followed by “serum starvation” in phenol red-free IMEM (Corning, Corning, NY, USA) containing 5% DCC-FBS and 1% P/S for 48 h. The cells were treated with either EtOH (vehicle control) or increasing concentrations of AnAc 24:1n5 for 48 h. MTT assays were performed with Cell Titer Reagent (Promega, Madison, WI, USA) according to the manufacturer’s instructions. For the FluoReporter™ Blue Fluorometric dsDNA Quantification Assay, the manufacturer’s instructions were followed. Three to four biological replicate experiments were performed for MTT and dsDNA Quant assays for each cell line. Within each experiment, quadruplicate technical replicates were performed. These data were analyzed using nonlinear regression in GraphPad Prism to estimate the IC_50_ values in Table 2.

## Figures and Tables

**Table 1:** Triple-negative breast cancer (TNBC) cell lines.

TNBC Cell Line	ATCC Cat. No.	Comments: Patient Source, Subtype, Mutations [22,23,24,25,26]	Medium (+1% Pen/Strep)
MDA-MB-231	HTB-26	White, basal B, MSL, claudin-low, p53 mut, BRCA WT, WT PI3K; KRAS G13D [27]; non-LAR (AR-negative)	DMEM with 10% FBS [28]
MDA-MB-468	HTB-132	Black, basal A, mesenchymal stem-like (MSL), p53 mutated, BRCA WT, PTEN homo deletion; EGFR amplification	DMEM with 10% FBS [29]
HCC1806	CRL-2335	Black, primary tumor: squamous carcinoma, basal A, basal-like BL2, p53 mut, BRCA WT, non-LAR (AR-negative)	RPMI 1640 with 10% FBS [30]
BT-20	HTB-19	White, primary tumor: adenocarcinoma, basal A, p53 mutated, BRCA WT, EGFR amplification; PI3CAmut PIK3CA H1047R [27]; RB1 mutated, non-LAR (AR-negative)	MEM with 10% FBS [29]

Abbreviations Table 1: DMEM = Dulbecco’s Modified Eagle Medium; FBS = fetal bovine serum; LAR = luminal androgen receptor; MEM = Minimum Essential Medium; mut = mutation; RPMI = Roswell Park Memorial Institute; WT = wild type.

**Table 2:** Anacardic acid (AnAc) IC_50_ values on BC cell lines. The IC_50_ values for AnAc were determined by MTT and dsDNA quantification assays and were calculated from 3–4 separate experiments. The IC_50_ values were calculated by nonlinear regression in GraphPad Prism. For the metabolomics analysis, cell lines were treated with indicated AnAc concentrations for 24 h.

Cell Line	AnAc Cell Viability (MTT) 48 h IC_50_ µM	AnAc Cell Proliferation (dsDNA Quantification) 48 h IC_50_ µM	µM AnAc 24 h Treatment for Metabolomics
MCF-7	21 ± 6.1 × 10^−6^	38 ± 4.4 × 10^−6^	28
MDA-MB-231	38 ± 5.6 × 10^−6^	30 ± 4.3 × 10^−6^	20
MDA-MB-468	39 ± 2.2 × 10^−6^	60 ± 8.4 × 10^−6^	20
HCC1806	41 ± 6.5 × 10^−6^	48 ±6.2 × 10^−6^	27
BT-20	59 ± 5.8 × 10^−6^	79 ± 1.0 × 10^−6^	40

**Table 3:** Metabolites significantly altered by AnAc treatment (vs. EtOH control) of BC cells. Statistical evaluation was performed in MetaboAnalyst 6.0 with data displayed according to *p* values.

Cells	AnAc-Altered Metabolites	Log2(FC)	*p* Value
MCF-7	ribulose-5-phosphate	−1.5615	0.0019888
	uracil	−1.9264	0.0022824
	xanthosine	−1.3499	0.0030643
	gluconic acid	−1.6437	0.0038966
	ethanolamine	−1.2497	0.0063086
	cystine (DL-cystine)	−1.1738	0.0080923
	ribose-5-phosphate	−2.0159	0.0084623
	galactinol	1.379	0.014507
	glucose-6-phosphate	−1.2953	0.014769
	cytidine	−1.1586	0.017964
	pseudo uridine	−1.0049	0.021683
	fructose-1-phosphate	−2.3767	0.02596
	docosahexaenoic acid (DHA)	−2.0523	0.027704
	5-methyluridine	−1.5322	0.031222
	thymine	−1.5992	0.035159
	alpha-aminoadipic acid	−1.1237	0.047021
	glucose	−1.0835	0.048064
HCC1806	octadecanol	2.5632	2.53 × 10^−5^
	1-hexadecanol	2.4413	0.00018262
	glucose-1-phosphate	−1.4218	0.0032117
	putrescine	−1.2587	0.019973
	azelaic acid	−1.0126	0.020676
	maltotriose	−1.2791	0.050363
BT-20	UDP-glucuronic acid	1.7788	2.81 × 10^−5^
	1-hexadecanol	3.0591	0.00018873
	citric acid	−1.0428	0.014918
	glutamine	−1.0999	0.01615
	cystine (DL-cystine)	1.738	0.033439
	galactinol	1.5808	0.042584
MDA-MB-231	aspartic acid	0.5855	0.0015669
	phytosphingosine	−0.67158	0.0016425
	sinigrin	−1.6591	0.0022074
	lanosterol	−1.1452	0.0022373
	4-hydroxybutyric acid	−0.70683	0.003666
	dehydrocholecalciferol (24,25-dihydroxyvitamin D)	−1.2079	0.01005
	octadecylglycerol	−0.86493	0.013566
	cholesterone	−0.68607	0.01592
	inositol-4-monophosphate	−0.94447	0.019321
	pyrophosphate	0.95923	0.03129
	xylonic acid	−0.73111	0.034291
	lactose	−1.7446	0.036909
MDA-MB-468	1-hexadecanol	1.3145	0.00030833
	glucuronic acid	0.60653	0.0069837
	xylitol	0.60827	0.034831
	D-erythro-sphingosine	−0.59322	0.036251

**Table 4:** Integrated metabolomics and transcriptomics analysis for AnAc-treated MCF-7 cells. Enrichment analysis by Pathway Maps in MetaCore of AnAc-regulated differential metabolites (VIP > 1.7, *p* < 0.05) in MCF-7 cells (24 h treatment with 28 μM AnAc) integrated with significant AnAc-regulated changes in mRNA transcript abundance (differentially expressed genes, DEGs) identified previously in MCF-7 cells with a *p* value cut off < 0.05 (6 h treatment with 13.5 µM AnAc, GEO GSE78011) [19]. Min = minimum; FDR = false discovery rate; In Data = the number of metabolites or DEGs in AnAc-treated MCF-7 cells that are in the Pathway Map pathway indicated.

			Metabolomics				RNA-Seq (DEGs)			
Pathway Maps	Min (*p* Value)	Min FDR	*p*-Value	FDR	In Data	Network Objects from Active Data	*p*-Value	FDR	In Data	Network Objects from Active Data
Regulation of lipid metabolism_Fatty acid-dependent regulation of lipidic metabolism	3.4 × 10^−3^	6.6 × 10^−2^	8.1 × 10^−2^	1.5 × 10^−1^	1	Linoleic acid	3.4 × 10^−3^	6.6 × 10^−2^	2	INSIG, PDK4
Signal transduction_mTORC1 downstream signaling	1.1 × 10^−1^	1.5 × 10^−1^	1.1 × 10^−1^	1.5 × 10^−1^	1	D-Ribose 5-phosphate	1.1 × 10^−1^	1.5 × 10^−1^	1	SCD

**Table 5:** Integrated metabolomics and transcriptomics analysis for AnAc-treated MDA-MB-231 cells. Enrichment analysis by Pathway Maps in MetaCore integrated AnAc-regulated differential metabolites (VIP > 1.7, *p* < 0.05) in MDA-MB-231 cells (24 h treatment with 20 μM AnAc) with significant AnAc-regulated changes in mRNA transcript abundance (differentially expressed genes, DEGs) identified previously in MDA-MB-231 cells with a *p* value cutoff < 0.05 (6 h treatment with 35 µM AnAc, GEO GSE78011) [19]. Min = minimum, FDR = false discovery rate, In Data = the number of metabolites or DEGs in AnAc-treated MDA-MB-231 cells that are in the Pathway Map pathway indicated.

			Metabolomics				RNA-Seq (DEGs)			
Pathway Maps	Min (*p* Value)	Min FDR	*p*-Value	FDR	In Data	Network Objects from Active Data	*p*-Value	FDR	In Data	Network Objects from Active Data
Signal transduction_ Amino acid-dependent mTORC1 activation	2.3 × 10^−6^	8.9 × 10^−5^	2.3 × 10^−6^	8.9 × 10^−5^	4	L-Leucine	0.13	0.28	5	SESN2 (Sestrin 2), SLC3A2, SLC1A5 (ASCT2), IPMK, SLC38A2
Mechanisms of drug resistance in multiple myeloma	1.0 × 10^−4^	2.7 × 10^−3^	4.7 × 10^−2^	7.4 × 10^−2^	1	UDP-D-glucuronic acid cytoplasm	1.0 × 10^−4^	2.7 × 10^−3^	8	IL-6, HES1, IL-8, MYC, CCND1 (Cyclin D1), HRAS, TCF7L2 (TCF4), FZD2 (Frizzled)
Urea cycle	6.3 × 10^−4^	9.4 × 10^−3^	6.3 × 10^−4^	9.4 × 10^−3^	2	L-Aspartic acid cytoplasm, Fumaric acid intracellular	0.64	0.751	1	AQP3 (Aquaporin 3)
Immune response_ Distinct metabolic pathways in naive and effector CD8+ T cells	2.6 × 10^−3^	1.7 × 10^−2^	2.6 × 10^−3^	1.7 × 10^−2^	2	L-Leucine	4.6 × 10^−3^	3.3 × 10^−2^	7	4E-BP1, MYC, SLC3A2, SLC1A5, LCK, SLC38A1, SLC38A2
Aminoacyl-tRNA biosynthesis in mitochondrion	3.1 × 10^-^	1.7 × 10^−2^	3.1 × 10^-^	1.7 × 10^−2^	2	L-Aspartic acid cytoplasm, L-Leucine cytoplasm	0.90	0.90	1	GARS1
Aminoacyl-tRNA biosynthesis in cytoplasm	4.5 × 10^−3^	2.1 × 10^−2^	4.5 × 10^-^	2.1 × 10^−2^	2	L-Aspartic acid cytoplasm, L-Leucine cytoplasm	0.93	0.93	1	GARS1
Neurophysiological process_Role of CDK5 in presynaptic signaling	2.9 × 10^−2^	7.5 × 10^−2^	2.9 × 10^−2^	7.53 × 10^−2^	1	Inositol 4-phosphate intracellular	0.55	0.7	1	SYT1 (Synaptotagmin I)
Prolactin signaling in Prostate Cancer	3.4 × 10^−2^	7.5 × 10^−2^	3.4 × 10^−2^	7.5 × 10^−2^	1	L-Aspartic acid cytoplasm	0.61	0.73	1	CCND1 (Cyclin D1)
Regulation of CFTR gating (normal and CF)	3.5 × 10^−2^	7.5 × 10^−2^	3.5 × 10^−2^	7.5 × 10^−2^	1	Pyrophosphate cytoplasm	0.62	0.74	1	PDE4D
Development_Thrombospondin 1 signaling	3.7 × 10^−2^	7.5 × 10^−2^	3.7 × 10^−2^	7.5 × 10^−2^	1	Pyrophosphate cytoplasm	7.9 × 10^−2^	0.19	3	VLDLR, VEGF-A, VEGFR-2
Dysregulation of Adiponectin secretion from adipocytes in obesity, type 2 diabetes and metabolic syndrome X	3.9 × 10^−2^	7.5 × 10^−2^	3.9 × 10^−2^	7.5 × 10^−2^	1	Fumaric acid intracellular	0.66	0.76	1	FKHR
Neurophysiological process_Glucose-excited neurons of arcuate nucleus (rodent model)	4.1 × 10^−2^	7.5 × 10^−2^	4.1 × 10^−2^	7.5 × 10^−2^	1	Fumaric acid intracellular	0.68	0.77	1	GLUT3
Neurophysiological process_Glucose-inhibited neurons of ventromedial and arcuate nuclei	4.2 × 10^−2^	7.5 × 10^−2^	4.2 × 10^−2^	7.5 × 10^−2^	1	Pyrophosphate cytoplasm	0.68	0.771	1	GLUT3
Sulfur metabolism	4.3 × 10^−2^	7.5 × 10^−2^	4.3 × 10^−2^	7.5 × 10^−2^	1	L-Cystathionine intracellular	0.69	0.77	1	CTH
Disruption of methionine metabolism in induction and progression of HCC	4.3 × 10^−2^	7.5 × 10^−2^	4.3 × 10^−2^	7.5 × 10^−2^	1	L-Cystathionine intracellular	0.69	0.771	1	c-Myc
L-Alanine and L-cysteine metabolism	4.7 × 10^−2^	7.5 × 10^−2^	4.7 × 10^−2^2	7.5 × 10^−2^	1	L-Cystathionine intracellular	0.37	0.541	2	CTH, GPT2
Neurophysiological process_Circadian rhythm	4.8 × 10^−2^	7.5 × 10^−2^	4.8 × 10^−2^	7.53 × 10^−2^	1	Pyrophosphate cytoplasm	0.73	0.79	1	REV-ERBalpha (NR1D1)
Metabolism of L-cysteine, D-cysteine and L-cystine	5.0 × 10^−2^	0.11	10.11	0.11	1	L-Cystathionine intracellular	5.0 × 10^−2^	0.15	7	ASCT1 (SLC1A4), CTH, SLC3A2, SLC38A4, SLC7A11, SLC38A1, SLC38A2

## Data Availability

RNA-seq data used in this report are from our analysis that is available at GEO: accession number GSE78011. Supplementary Table S1 includes the raw data from West Coast Metabolomics.

## Supplementary Materials

The following supporting information can be downloaded at https://www.mdpi.com/article/10.3390/ijms25137044/s1.
