# Supplementary material for: Integrated Metabolomics and Transcriptomics Analysis of Anacardic Acid Inhibition of Breast Cancer Cell Viability
Source: Int J Mol Sci. 2024 Jun 27;25(13):7044. doi: 10.3390/ijms25137044 (PMC11241071; doi:10.3390/ijms25137044)
Supplement: Supplementary file 1 [file ijms-25-07044-s001.zip › ijms-3005065-Supplementary caption.pdf]

Supplementary Files for

Integrated Metabolomics and Transcriptomics Analysis of Anacardic Acid Inhibition of Breast Cancer Cell Viability

Kellianne M. Piell[1], Claire C. Poulton[1], David J. Schultz[2], Christian G. Stanley[1], and Carolyn M. Klinge[1*]

[1]Department of Biochemistry & Molecular Genetics, University of Louisville School of Medicine;

[2] Department of Biology, University of Louisville; Louisville, KY 40292 USA

*Corresponding author: Carolyn M. Klinge, Department of Biochemistry & Molecular Genetics University of Louisville School of Medicine, Louisville, KY

+++

Three Supplementary Tables:

1. Supplementary Table 1 = Excel file with West Coast Metabolomics data
2. Supplementary Table 2 = SAM (significance of metabolites) analysis identified 96 metabolites in EtOH-treated cells.
3. Supplementary Table 3 = SAM (significance of metabolites) analysis identified 128 metabolites in AnAc-treated cells.
4. Supplementary Table 4A = Top 10 enrichment by pathway maps for metabolites identified as significantly altered by AnAc (versus EtOH) in MCF-7 cells inMetaCore.
5. Supplementary Table 4B = op 10 pathway maps for metabolites identified as significantly altered by AnAc (versus EtOH) in MCF-7 cells in MetaboAnalyst Volcano Plots.
6. Supplementary Table 5A = Top 10 enrichment by pathway maps for metabolites identified as significantly altered by AnAc (versus EtOH) in HCC1806 cells in MetaCore.
7. Supplementary Table 5B = Six pathway maps for metabolites identified as significantly altered by AnAc (versus EtOH) in HCC1806 cells in MetaboAnalyst Volcano Plot as identified in MetaCore.
8. Supplementary Table 6A = Top 10 enrichment by pathway maps for metabolites identified as significantly altered by AnAc (versus EtOH) in BT-20 cells inMetaCore.
9. Supplementary Table 6B = Top 10 enrichment by pathway maps for metabolites identified as significantly altered by AnAc (versus EtOH) in BT-20 cells in MetaboAnalyst Volcano Plots (Supplementary Table 5).
10. Supplementary Table 7A = Top 10 enrichment by pathway maps for metabolites identified as significantly altered by AnAc (versus EtOH) in MDA-MB-2310 cells in MetaCore.
11. Supplementary Table 7B = Top 10 enrichment by pathway maps for metabolites identified as significantly altered by AnAc (versus EtOH) in MDA-MB-231 cells in MetaboAnalyst Volcano Plot 1.5 as identified in MetaCore.

12. Supplementary Table 8A = Top 10 enrichment by pathway maps for metabolites identified as significantly altered by AnAc (versus EtOH) in MDA-MB-468 cells in MetaCore.
13. Supplementary Table 8B = Top 10 enrichment by pathway maps for metabolites identified as significantly altered by AnAc (versus EtOH) in MDA-MB-468 cells in MetaboAnalyst Volcano Plot 1.5 as identified in MetaCore.

Supplementary Figures

- Supplementary Figure 1: AnAc inhibits the cell proliferation of ER+ and TNBC cells.
- Supplementary Figure 2: PCA analysis of all identified metabolites from the control and AnAc-treated cell lines.
- Supplementary Figure 3: Heatmap of all identified metabolites from the control (EtOH) and AnAc treated cell lines.
- Supplementary Figure 4: Variable importance (VIP) of top 15 metabolites identified in OPLS-DA of metabolites from the control (EtOH) and AnAc-treated BC cell lines in MetaboAnalyst 6.0.
- Supplementary Figure 5: VIP-plots and Enrichment Analysis of the top 15 metabolites identified in OPLS-DA of metabolites from the control (EtOH) and AnAc-treated MCF-7 and HCC1806 cell lines analyzed by MetaboAnalyst 6.0.
- Supplementary Figure 6: VIP-plots of the top 15 metabolites identified in OPLS-DA of metabolites from the control (EtOH) and AnAc-treated BT-20, MDA-MB-231, and MDA-MB-468 cell lines analyzed by MetaboAnalyst 6.0.
- Supplementary Figure 7: Enrichment Analysis of metabolites significantly altered by AnAc treatment of BT-20, MDA-MB-231, and MDA-MB-468 cell lines analyzed by MetaboAnalyst 6.0 software.
- Supplementary Figure 8: Venn diagram of Pathway Maps for AnAc-regulated metabolites in the five BC cell lines.
- Supplementary Figure 9A and B: Top 2 Pathway Maps for metabolites in AnAc-treated MCF-7 cells with AnAc: A) “Signal transduction: Amino acid-dependent mTORC1 activation” and B) “Glycine and L-Serine metabolism”.
- Supplementary Figure 9C and D: Top 2 Pathway Maps for metabolites in MCF-7 cells treated with AnAc by MetaboAnalyst: C) “Immune response: The effect of IDO1 on T cell metabolism” and D) “Pentose phosphate pathway”.
- Supplementary Figure 10A and B: Top 2 Pathway Maps in HCC1806 cells treated with AnAc: A) “Glycogen metabolism” and B) “Transport_GPR40 signaling pathway in beta cells”.
- Supplementary Figure 10C and D: Top 2 Pathway Maps in HCC1806 cells treated with AnAc using metabolites identified as significant in Volcano Plots in MetaboAnalyst: C) “Glycogen metabolism” and D) “Immune response_IL-13 signaling via PI3K-ERK pathway”.
- Supplementary Figure 11A and B: Top 2 Pathway Maps in BT-20 cells treated with AnAc:
- Supplementary Figure 11C and D: Top 2 Pathway Maps in BT-20 cells treated with AnAc using metabolites identified as significant in Volcano Plots in MetaboAnalyst:
- Supplementary Figure 12A and B: Top 2 Pathway Maps for MDA-MB-231 cells treated with AnAc: A) “Signal transduction: Amino acid-dependent mTORC1 activation” and B) “Urea Cycle”.

- Supplementary Figure 12C and D: Top 2 Pathway Maps for MDA-MB-231 cells treated with AnAc using metabolites identified in Volcano Plots in MetaboAnalyst: C) “Neurophysiological process_Role of CDK5 in presynaptic signaling” and D) “Prolactin signaling in Prostate Cancer”.
- Supplementary Figure 13: Top 2 Pathway Maps MDA-MB-468 cells treated with AnAc : A) “Cholesterol and Sphingolipid transport” and B) “Cholesterol and Sphingolipid transport / Distribution to the intracellular membrane compartments”.
- Supplementary Figure 13C and D: Top 2 Pathway Maps MDA-MB-468 cells treated with AnAc using metabolites identified in Volcano Plots in MetaboAnalyst: C) “Signal transduction_Role of Sphingosine 1-phosphate as an intracellular mediator” and D) “Apoptosis and survival_Ubiquitination and phosphorylation in TNF-alpha-induced NF-kB signaling”.
- Supplementary Figure 14A and B: Pathway Maps from integrating metabolomics and RNA-seq analysis of AnAc-treated MCF-7 cells. A) “Regulation of lipid metabolism_Fatty acid-dependent regulation of lipidic metabolism”. B) “Signal transduction_mTORC1 downstream signaling”.
- Supplementary Figure 15: SCD (SCD1) protein abundance in control (EtOH) and AnAc-treated MDA-MB-231, MDA-MB-468, and MCF-7 BC cells.
- Supplementary Figure 16A and B: Top 2 Pathway Maps from integrating metabolomics and RNA-seq analysis of AnAc-treated MDA-MB-231 cells. A) “Signal transduction_Amino acid-dependent mTORC1 activation”. B) “Mechanisms of drug resistance in multiple myeloma”.
- Supplementary Figure 16C and D: Pathways 3 and 4 in Pathway Maps from integrating metabolomics and RNA-seq analysis of AnAc-treated MDA-MB-231 cells. C) “Urea Cycle”. C) “*De novo* IMP Biosynthesis”.
- Supplementary Figure 17: Flowchart of the experimental design.
