# Supplementary material for: Integrated Metabolomics and Transcriptomics Analysis of Anacardic Acid Inhibition of Breast Cancer Cell Viability
Source: Int J Mol Sci. 2024 Jun 27;25(13):7044. doi: 10.3390/ijms25137044 (PMC11241071; doi:10.3390/ijms25137044)
Supplement: Supplementary file 1 [file ijms-25-07044-s001.zip › ijms-3005065-supplementary figures.pptx]

## Slide 1
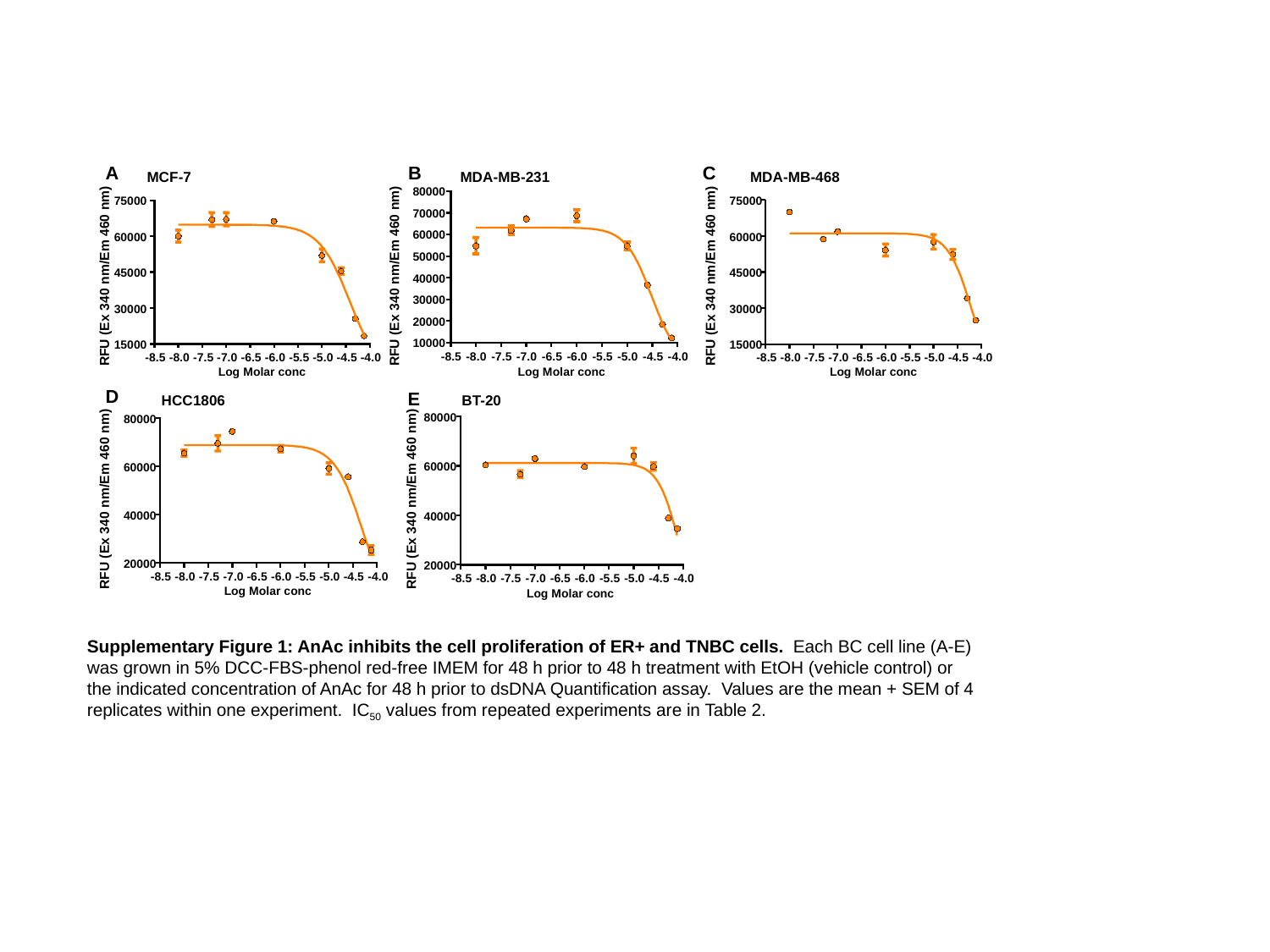

A
B
C
MCF-7
MDA-MB-231
MDA-MB-468
80000
75000
75000
70000
60000
60000
60000
50000
RFU (Ex 340 nm/Em 460 nm)
RFU (Ex 340 nm/Em 460 nm)
RFU (Ex 340 nm/Em 460 nm)
45000
45000
40000
30000
30000
30000
20000
10000
15000
15000
-8.5
-8.0
-7.5
-7.0
-6.5
-6.0
-5.5
-5.0
-4.5
-4.0
-8.5
-8.0
-7.5
-7.0
-6.5
-6.0
-5.5
-5.0
-4.5
-4.0
-8.5
-8.0
-7.5
-7.0
-6.5
-6.0
-5.5
-5.0
-4.5
-4.0
Log Molar conc
Log Molar conc
Log Molar conc
D
E
HCC1806
BT-20
80000
80000
60000
60000
RFU (Ex 340 nm/Em 460 nm)
RFU (Ex 340 nm/Em 460 nm)
40000
40000
20000
20000
-8.5
-8.0
-7.5
-7.0
-6.5
-6.0
-5.5
-5.0
-4.5
-4.0
-8.5
-8.0
-7.5
-7.0
-6.5
-6.0
-5.5
-5.0
-4.5
-4.0
Log Molar conc
Log Molar conc
Supplementary Figure 1: AnAc inhibits the cell proliferation of ER+ and TNBC cells. Each BC cell line (A-E) was grown in 5% DCC-FBS-phenol red-free IMEM for 48 h prior to 48 h treatment with EtOH (vehicle control) or the indicated concentration of AnAc for 48 h prior to dsDNA Quantification assay. Values are the mean + SEM of 4 replicates within one experiment. IC50 values from repeated experiments are in Table 2.

## Slide 2
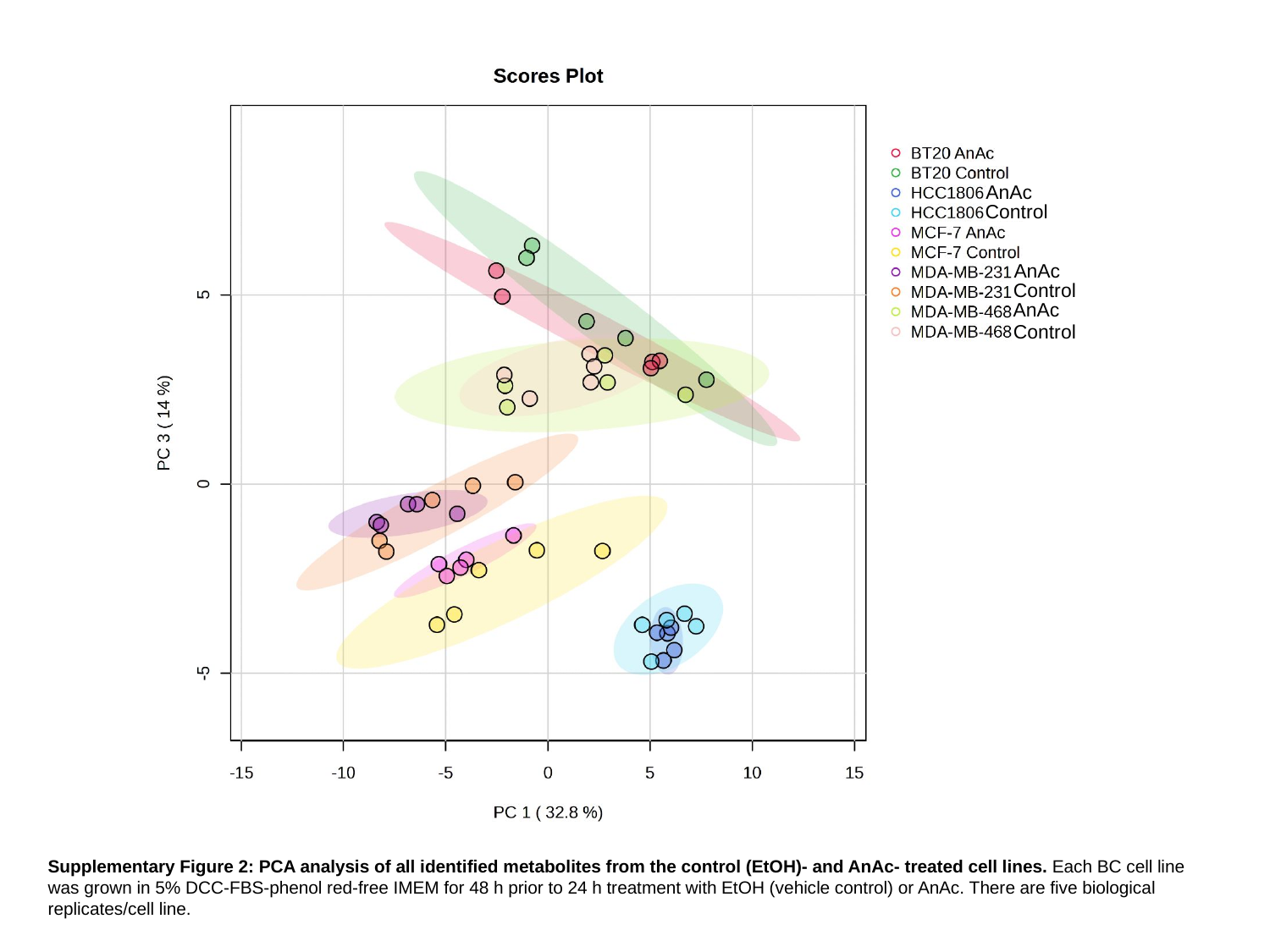

AnAc
Control
AnAc
Control
AnAc
Control
Supplementary Figure 2: PCA analysis of all identified metabolites from the control (EtOH)- and AnAc- treated cell lines. Each BC cell line was grown in 5% DCC-FBS-phenol red-free IMEM for 48 h prior to 24 h treatment with EtOH (vehicle control) or AnAc. There are five biological replicates/cell line.

## Slide 3
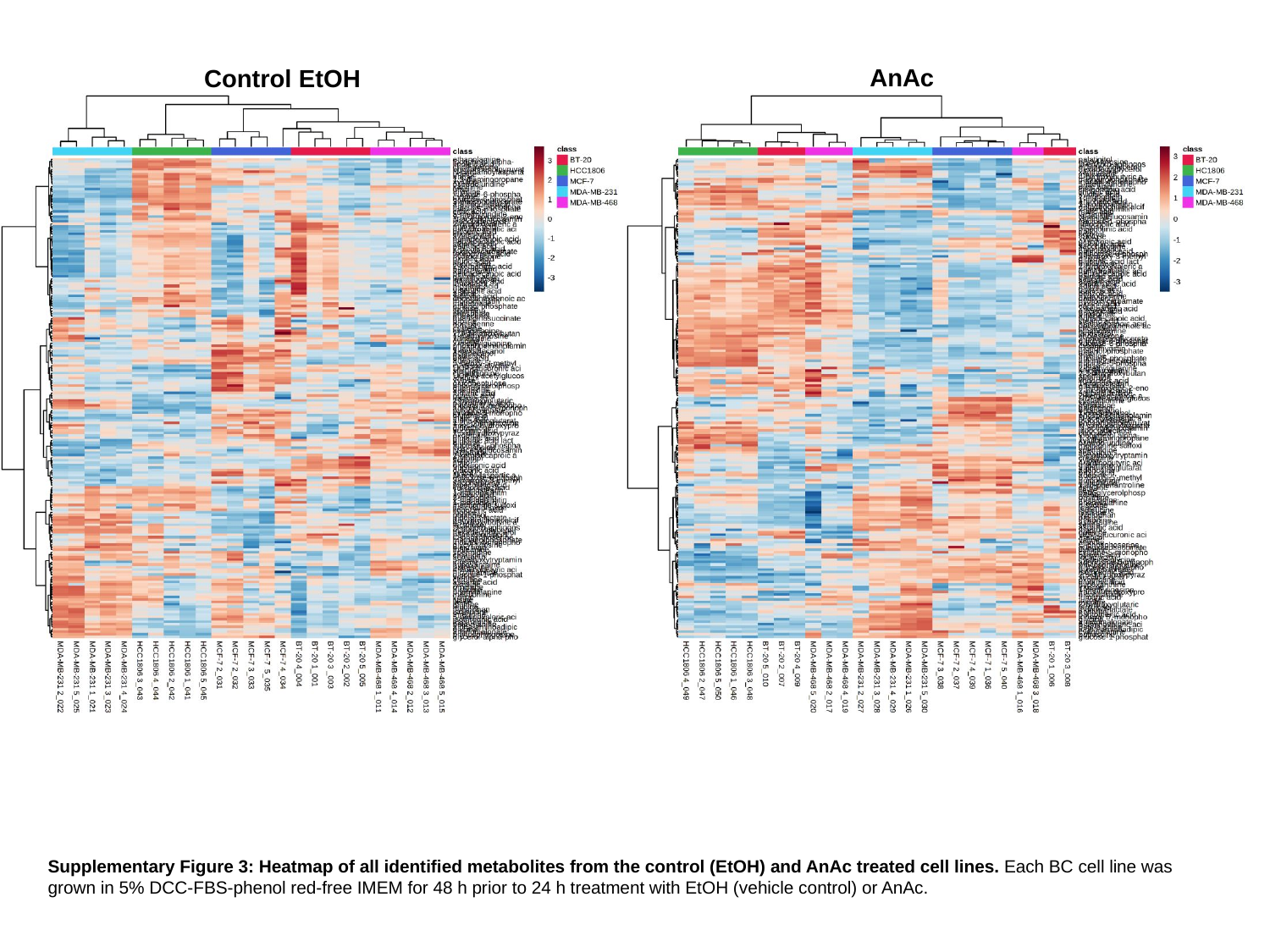

AnAc
Control EtOH
Supplementary Figure 3: Heatmap of all identified metabolites from the control (EtOH) and AnAc treated cell lines. Each BC cell line was grown in 5% DCC-FBS-phenol red-free IMEM for 48 h prior to 24 h treatment with EtOH (vehicle control) or AnAc.

## Slide 4
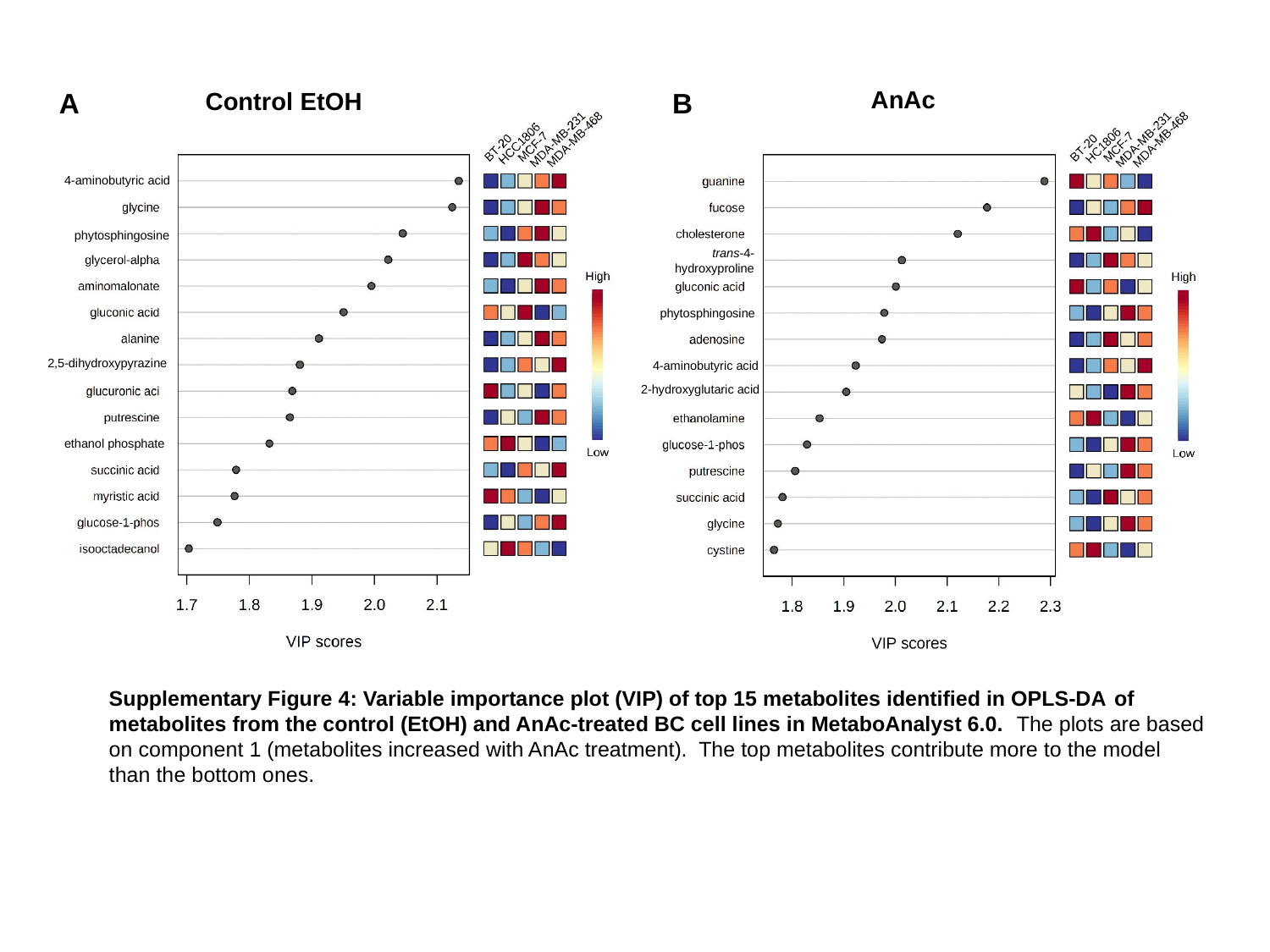

AnAc
Control EtOH
A
B
4-aminobutyric acid
phytosphingosine
trans-4-hydroxyproline
phytosphingosine
2,5-dihydroxypyrazine
4-aminobutyric acid
2-hydroxyglutaric acid
ethanol phosphate
Supplementary Figure 4: Variable importance plot (VIP) of top 15 metabolites identified in OPLS-DA of metabolites from the control (EtOH) and AnAc-treated BC cell lines in MetaboAnalyst 6.0. The plots are based on component 1 (metabolites increased with AnAc treatment). The top metabolites contribute more to the model than the bottom ones.

## Slide 5
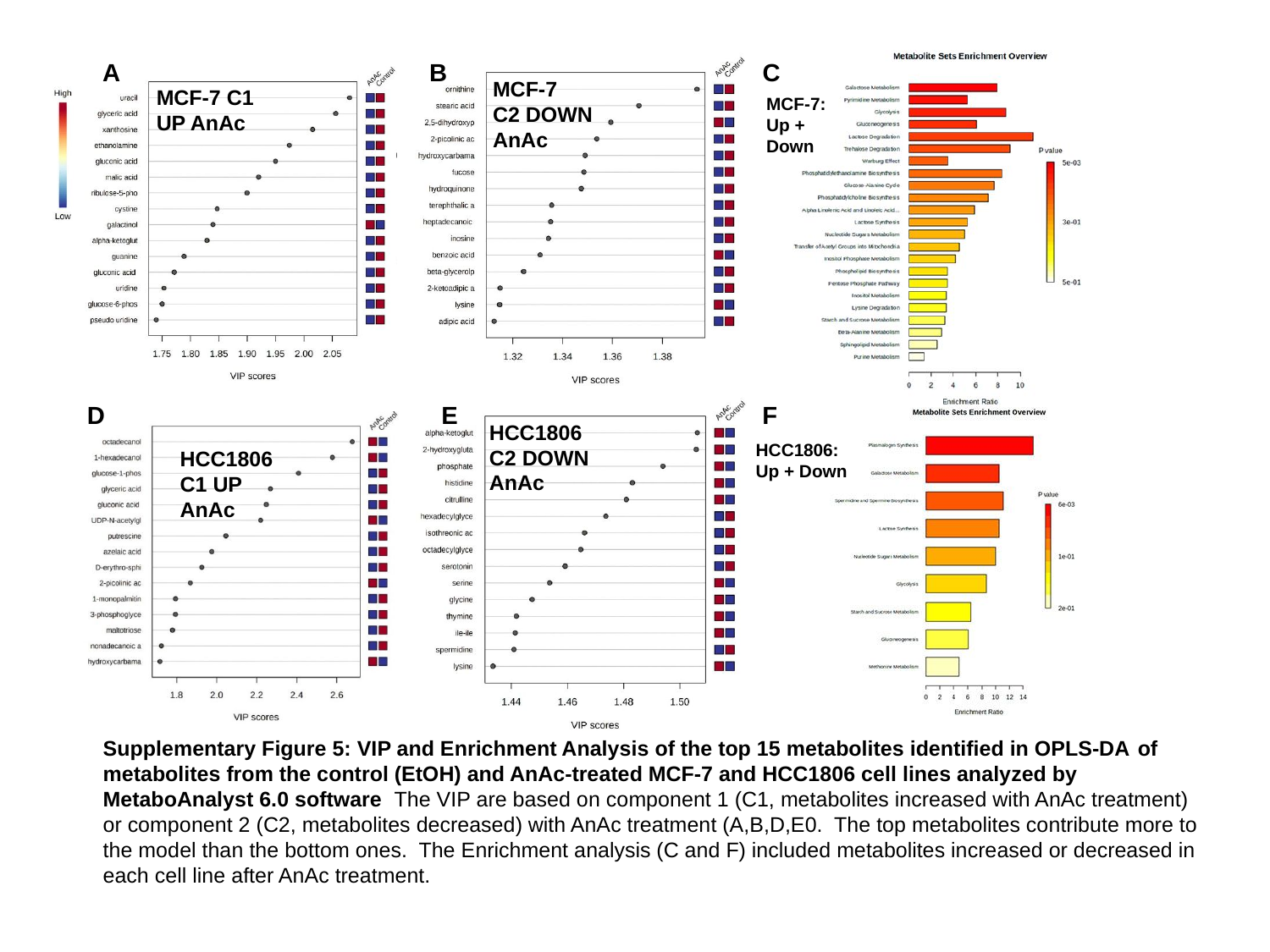

A
B
C
MCF-7
C2 DOWN
AnAc
MCF-7 C1 UP AnAc
MCF-7: Up + Down
D
E
F
HCC1806 C2 DOWN AnAc
HCC1806: Up + Down
HCC1806 C1 UP AnAc
Supplementary Figure 5: VIP and Enrichment Analysis of the top 15 metabolites identified in OPLS-DA of metabolites from the control (EtOH) and AnAc-treated MCF-7 and HCC1806 cell lines analyzed by MetaboAnalyst 6.0 software The VIP are based on component 1 (C1, metabolites increased with AnAc treatment) or component 2 (C2, metabolites decreased) with AnAc treatment (A,B,D,E0. The top metabolites contribute more to the model than the bottom ones. The Enrichment analysis (C and F) included metabolites increased or decreased in each cell line after AnAc treatment.

## Slide 6
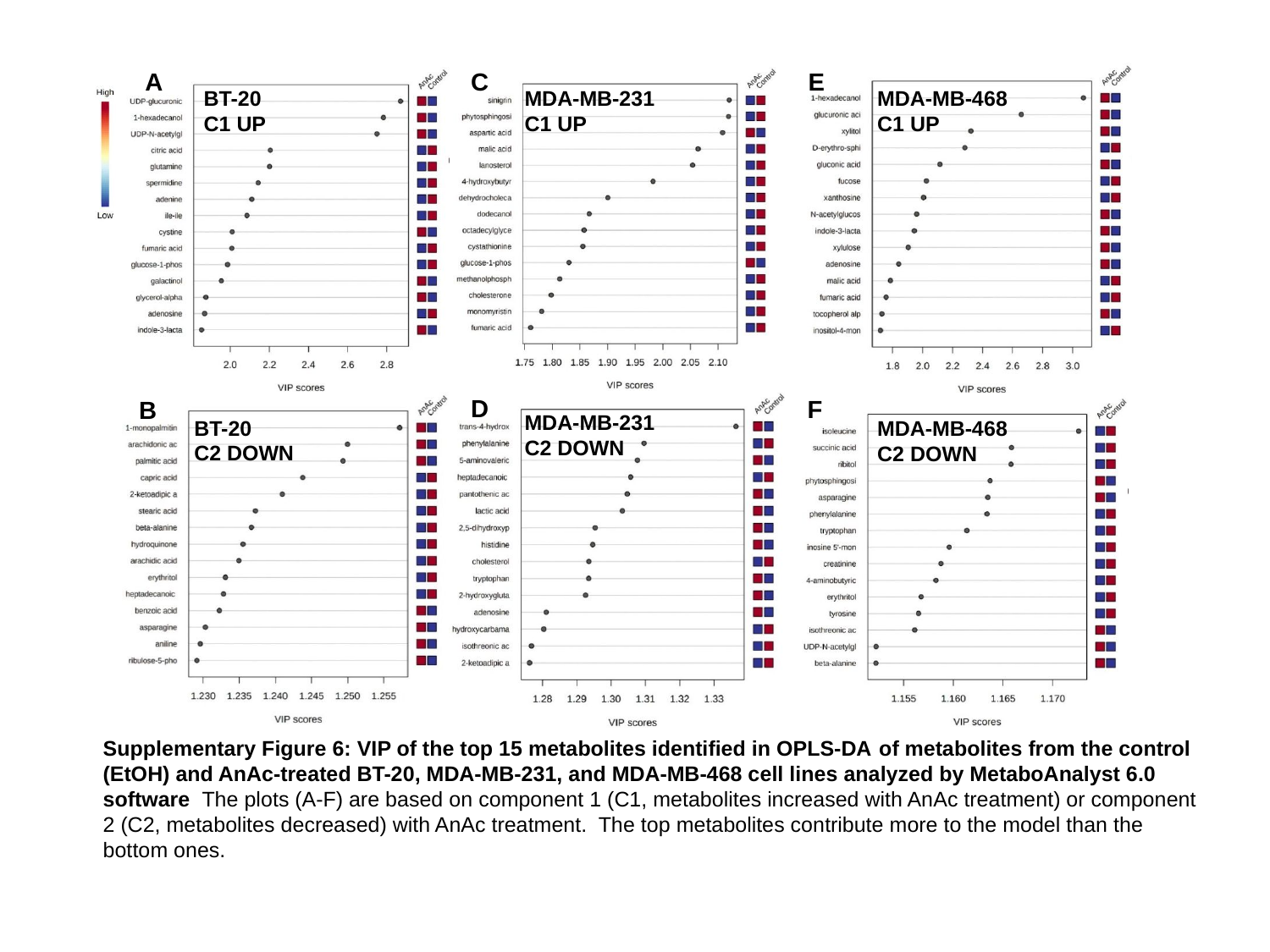

A
C
E
MDA-MB-468 C1 UP
BT-20
C1 UP
MDA-MB-231 C1 UP
D
F
B
MDA-MB-231 C2 DOWN
BT-20
C2 DOWN
MDA-MB-468 C2 DOWN
Supplementary Figure 6: VIP of the top 15 metabolites identified in OPLS-DA of metabolites from the control (EtOH) and AnAc-treated BT-20, MDA-MB-231, and MDA-MB-468 cell lines analyzed by MetaboAnalyst 6.0 software The plots (A-F) are based on component 1 (C1, metabolites increased with AnAc treatment) or component 2 (C2, metabolites decreased) with AnAc treatment. The top metabolites contribute more to the model than the bottom ones.

## Slide 7
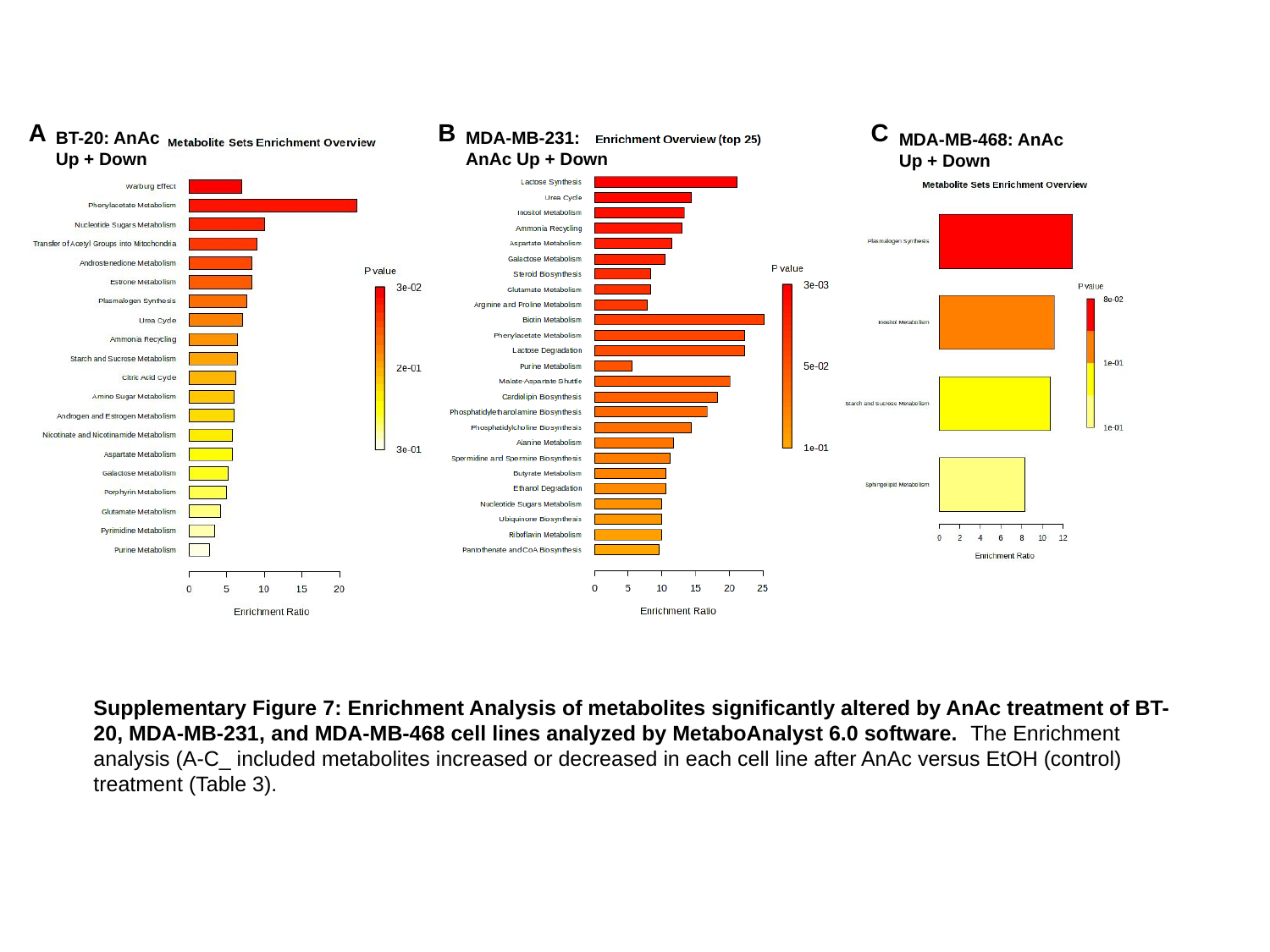

A
B
C
BT-20: AnAc Up + Down
MDA-MB-231: AnAc Up + Down
MDA-MB-468: AnAc Up + Down
Supplementary Figure 7: Enrichment Analysis of metabolites significantly altered by AnAc treatment of BT-20, MDA-MB-231, and MDA-MB-468 cell lines analyzed by MetaboAnalyst 6.0 software. The Enrichment analysis (A-C_ included metabolites increased or decreased in each cell line after AnAc versus EtOH (control) treatment (Table 3).

## Slide 8
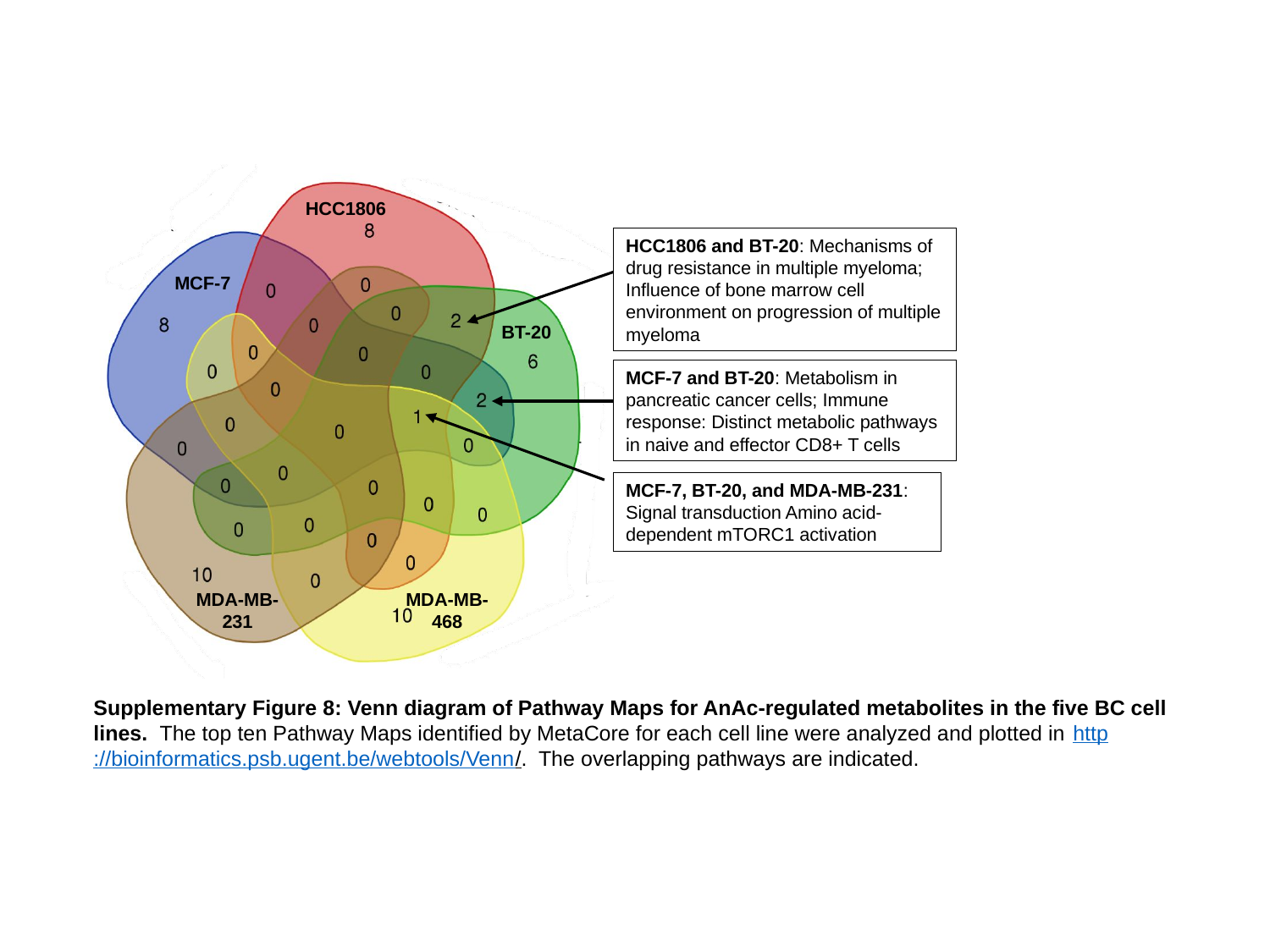

HCC1806
HCC1806 and BT-20: Mechanisms of drug resistance in multiple myeloma; Influence of bone marrow cell environment on progression of multiple myeloma
MCF-7
BT-20
MCF-7 and BT-20: Metabolism in pancreatic cancer cells; Immune response: Distinct metabolic pathways in naive and effector CD8+ T cells
MCF-7, BT-20, and MDA-MB-231: Signal transduction Amino acid-dependent mTORC1 activation
MDA-MB-231
MDA-MB-468
Supplementary Figure 8: Venn diagram of Pathway Maps for AnAc-regulated metabolites in the five BC cell lines. The top ten Pathway Maps identified by MetaCore for each cell line were analyzed and plotted in http://bioinformatics.psb.ugent.be/webtools/Venn/. The overlapping pathways are indicated.

## Slide 9
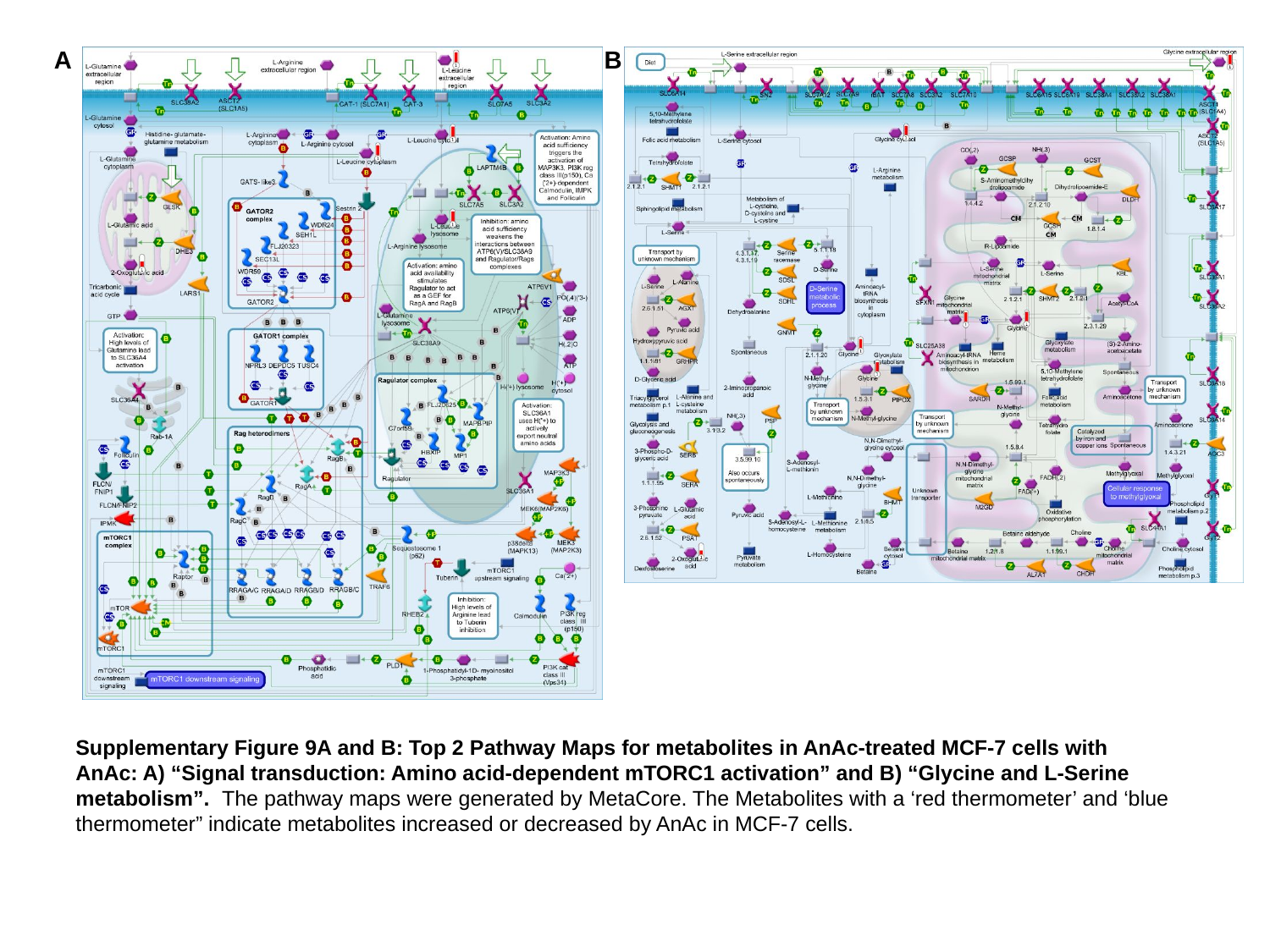

A
B
Supplementary Figure 9A and B: Top 2 Pathway Maps for metabolites in AnAc-treated MCF-7 cells with AnAc: A) “Signal transduction: Amino acid-dependent mTORC1 activation” and B) “Glycine and L-Serine metabolism”. The pathway maps were generated by MetaCore. The Metabolites with a ‘red thermometer’ and ‘blue thermometer” indicate metabolites increased or decreased by AnAc in MCF-7 cells.

## Slide 10
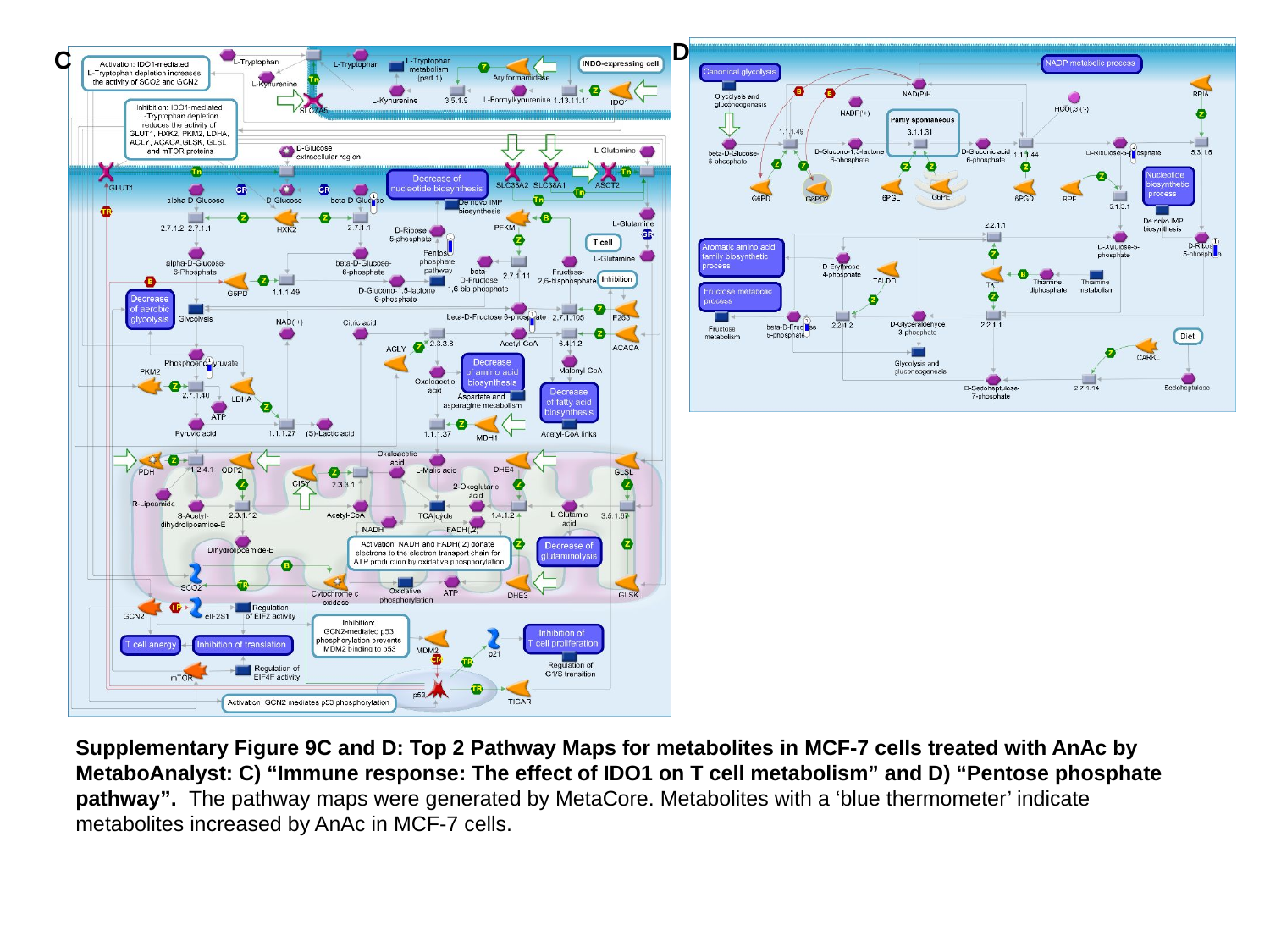

D
C
Supplementary Figure 9C and D: Top 2 Pathway Maps for metabolites in MCF-7 cells treated with AnAc by MetaboAnalyst: C) “Immune response: The effect of IDO1 on T cell metabolism” and D) “Pentose phosphate pathway”. The pathway maps were generated by MetaCore. Metabolites with a ‘blue thermometer’ indicate metabolites increased by AnAc in MCF-7 cells.

## Slide 11
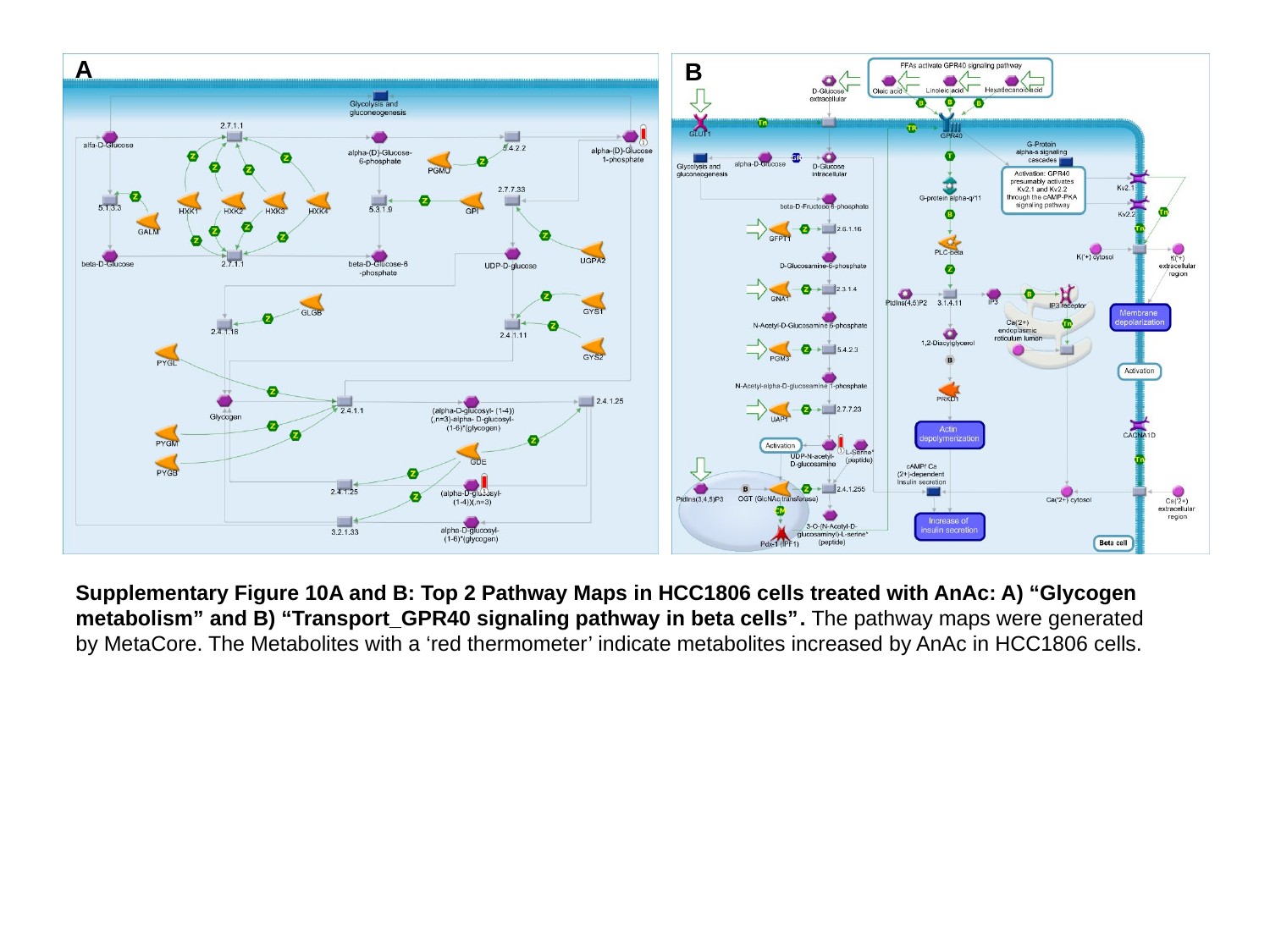

A
B
Supplementary Figure 10A and B: Top 2 Pathway Maps in HCC1806 cells treated with AnAc: A) “Glycogen metabolism” and B) “Transport_GPR40 signaling pathway in beta cells”. The pathway maps were generated by MetaCore. The Metabolites with a ‘red thermometer’ indicate metabolites increased by AnAc in HCC1806 cells.

## Slide 12
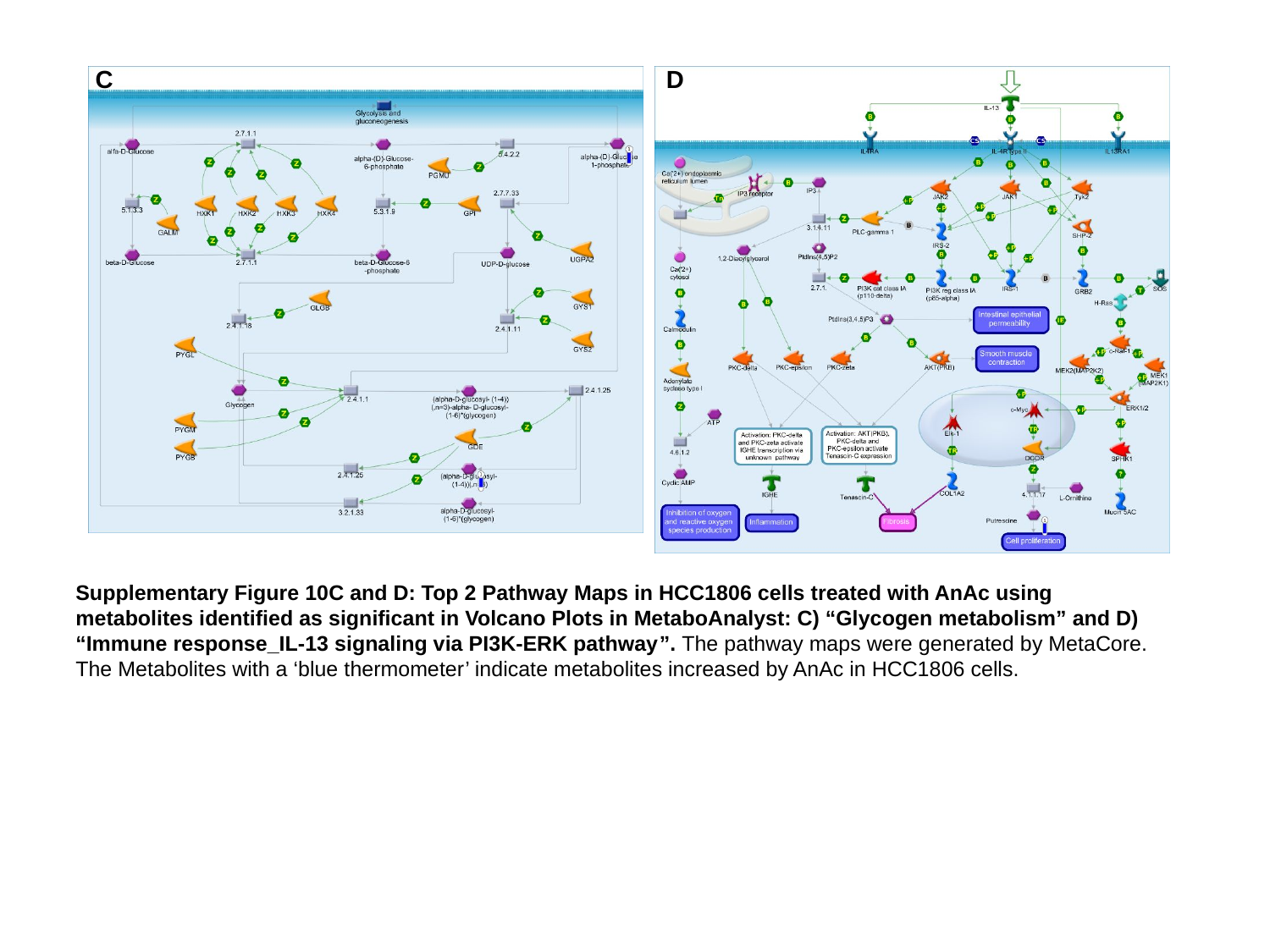

C
D
Supplementary Figure 10C and D: Top 2 Pathway Maps in HCC1806 cells treated with AnAc using metabolites identified as significant in Volcano Plots in MetaboAnalyst: C) “Glycogen metabolism” and D) “Immune response_IL-13 signaling via PI3K-ERK pathway”. The pathway maps were generated by MetaCore. The Metabolites with a ‘blue thermometer’ indicate metabolites increased by AnAc in HCC1806 cells.

## Slide 13
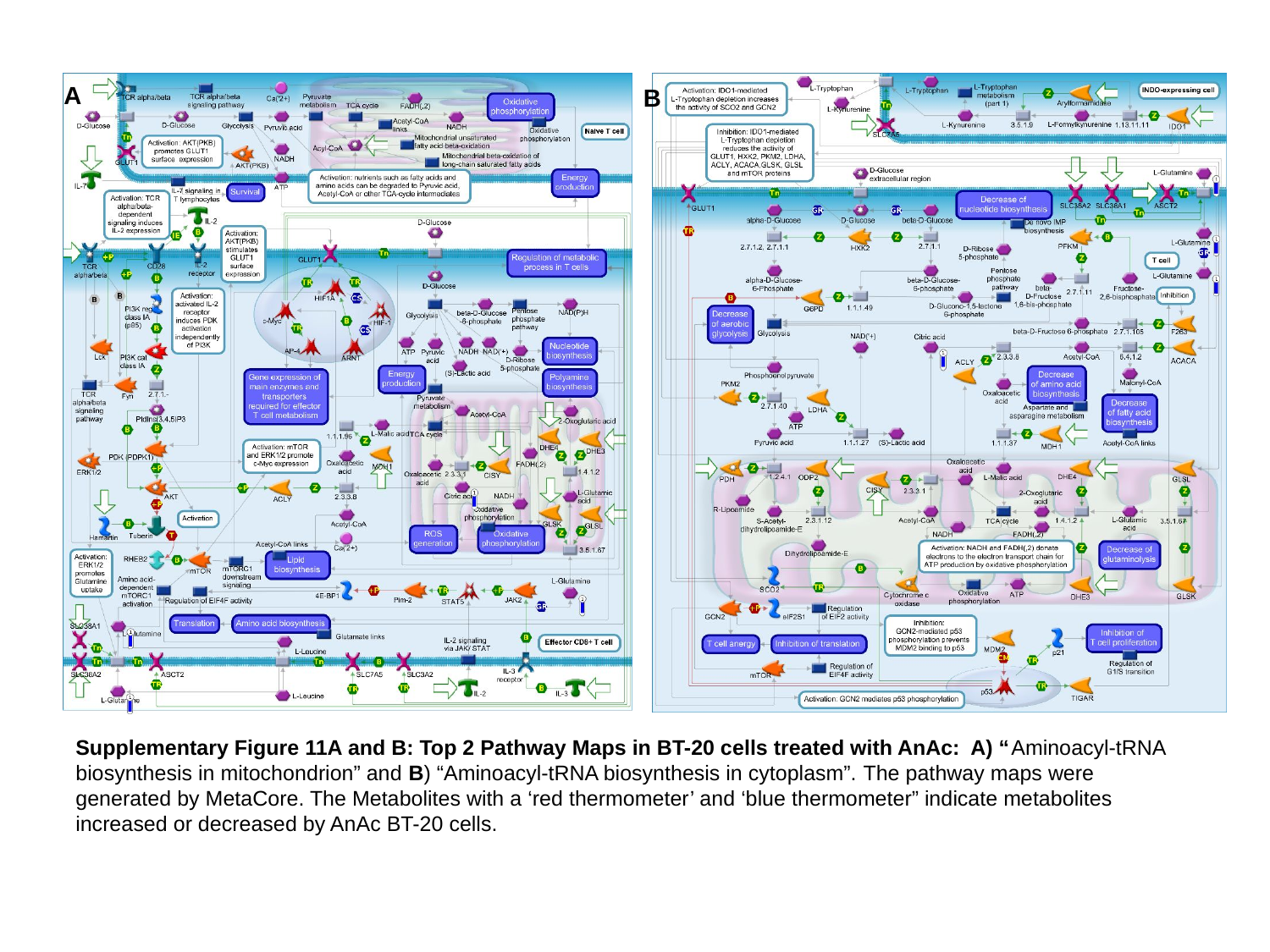

A
B
Supplementary Figure 11A and B: Top 2 Pathway Maps in BT-20 cells treated with AnAc: A) “Aminoacyl-tRNA biosynthesis in mitochondrion” and B) “Aminoacyl-tRNA biosynthesis in cytoplasm”. The pathway maps were generated by MetaCore. The Metabolites with a ‘red thermometer’ and ‘blue thermometer” indicate metabolites increased or decreased by AnAc BT-20 cells.

## Slide 14
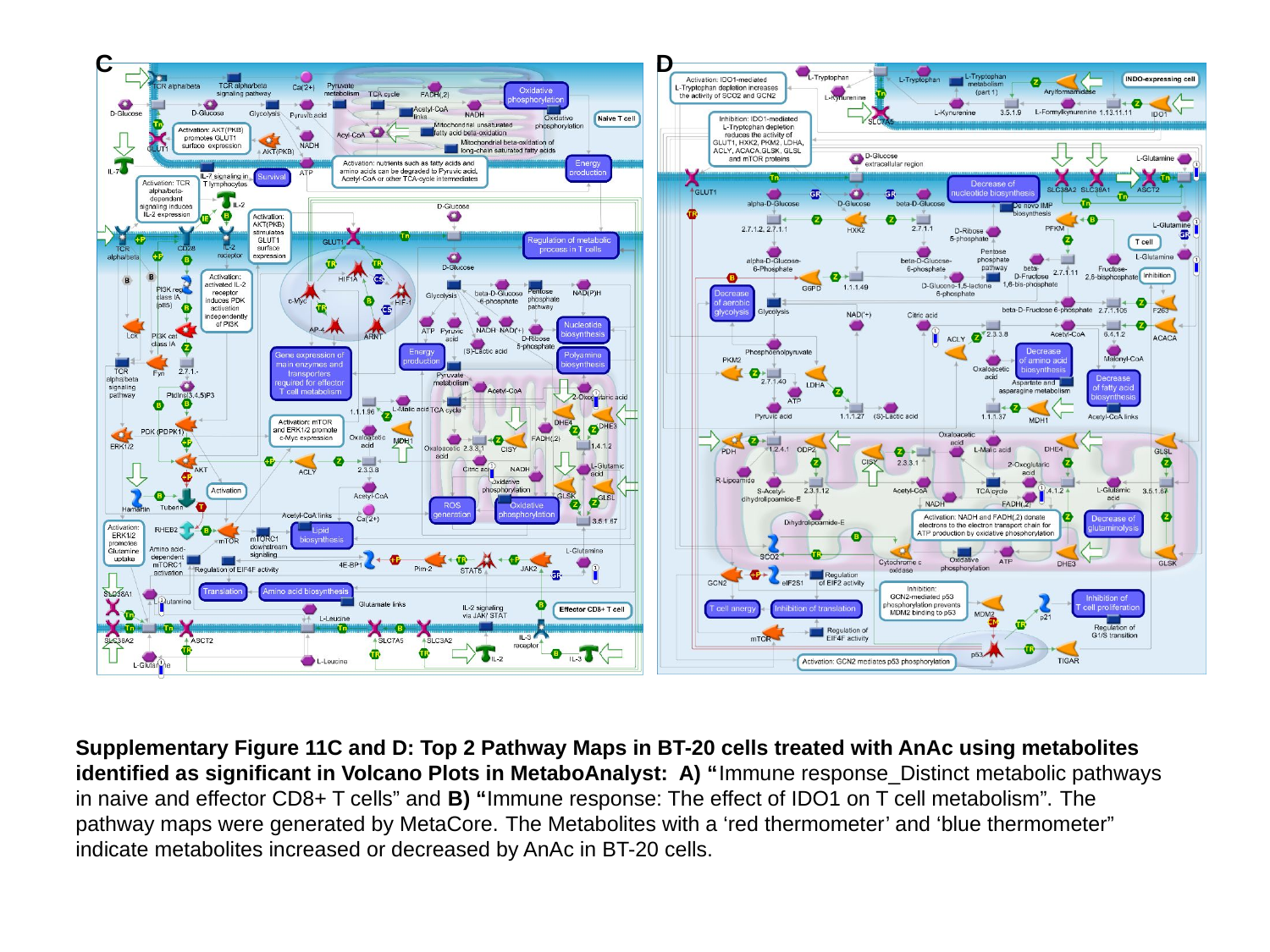

C
D
Supplementary Figure 11C and D: Top 2 Pathway Maps in BT-20 cells treated with AnAc using metabolites identified as significant in Volcano Plots in MetaboAnalyst: A) “Immune response_Distinct metabolic pathways in naive and effector CD8+ T cells” and B) “Immune response: The effect of IDO1 on T cell metabolism”. The pathway maps were generated by MetaCore. The Metabolites with a ‘red thermometer’ and ‘blue thermometer” indicate metabolites increased or decreased by AnAc in BT-20 cells.

## Slide 15
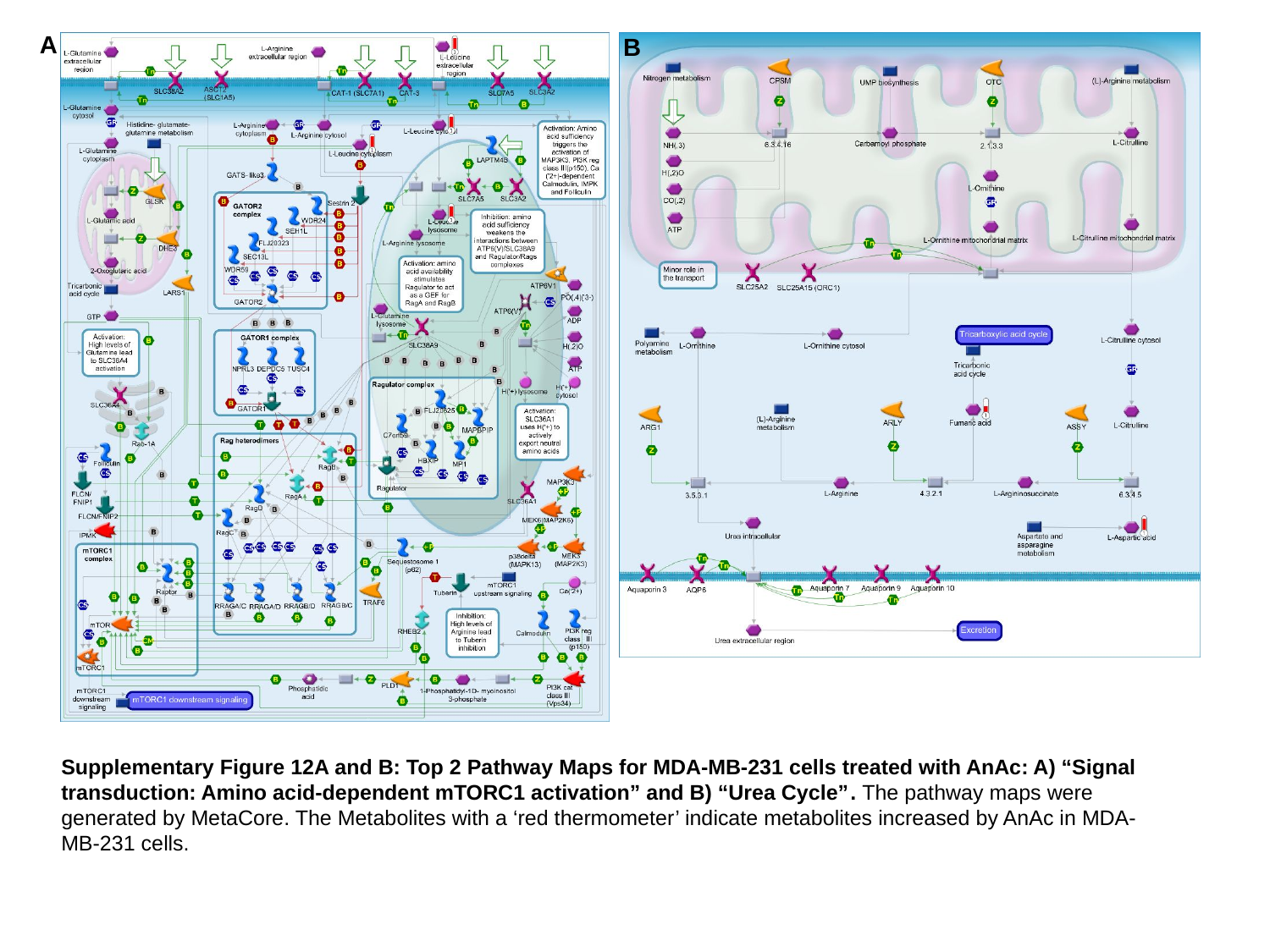

A
B
Supplementary Figure 12A and B: Top 2 Pathway Maps for MDA-MB-231 cells treated with AnAc: A) “Signal transduction: Amino acid-dependent mTORC1 activation” and B) “Urea Cycle”. The pathway maps were generated by MetaCore. The Metabolites with a ‘red thermometer’ indicate metabolites increased by AnAc in MDA-MB-231 cells.

## Slide 16
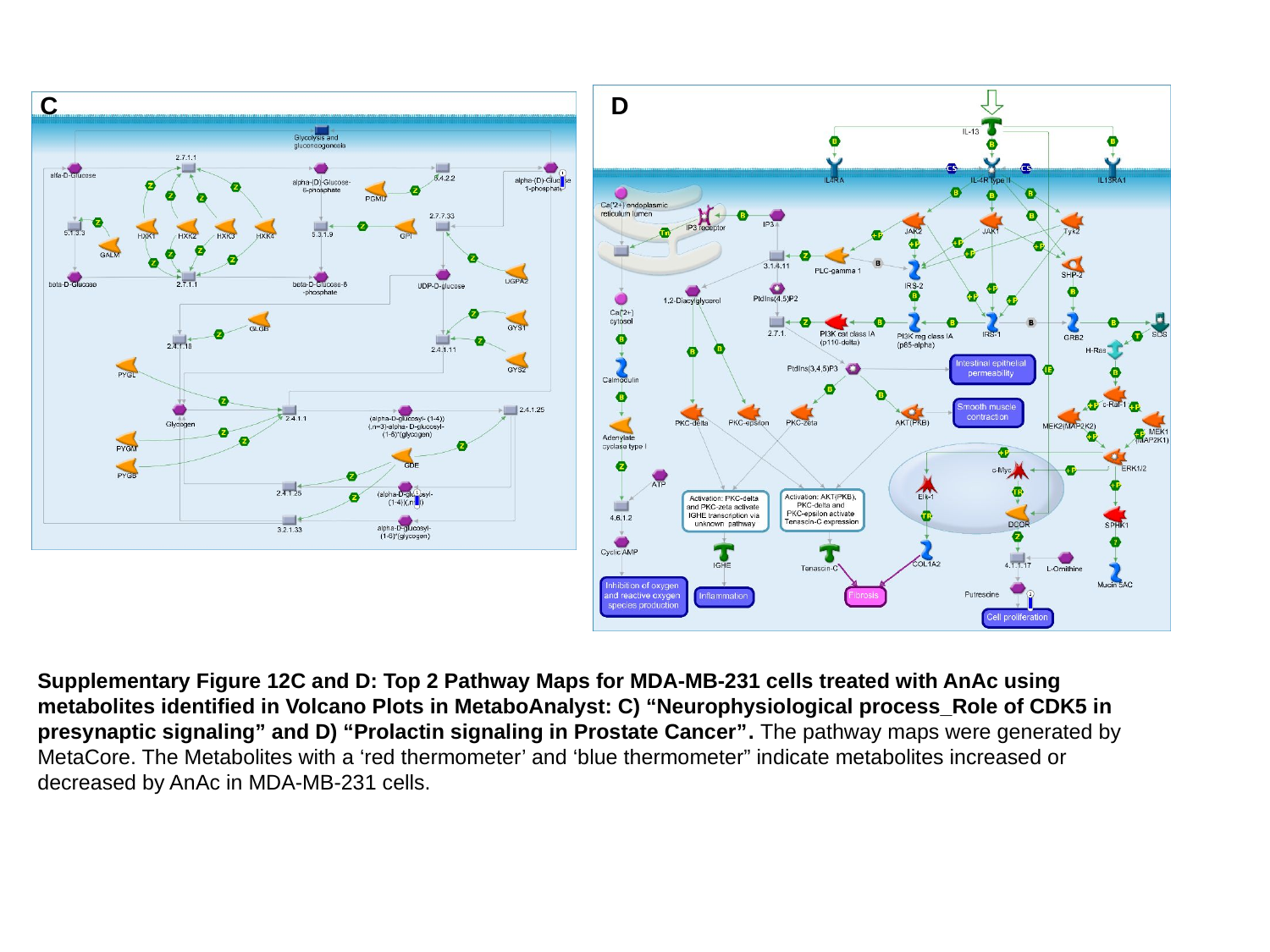

C
D
Supplementary Figure 12C and D: Top 2 Pathway Maps for MDA-MB-231 cells treated with AnAc using metabolites identified in Volcano Plots in MetaboAnalyst: C) “Neurophysiological process_Role of CDK5 in presynaptic signaling” and D) “Prolactin signaling in Prostate Cancer”. The pathway maps were generated by MetaCore. The Metabolites with a ‘red thermometer’ and ‘blue thermometer” indicate metabolites increased or decreased by AnAc in MDA-MB-231 cells.

## Slide 17
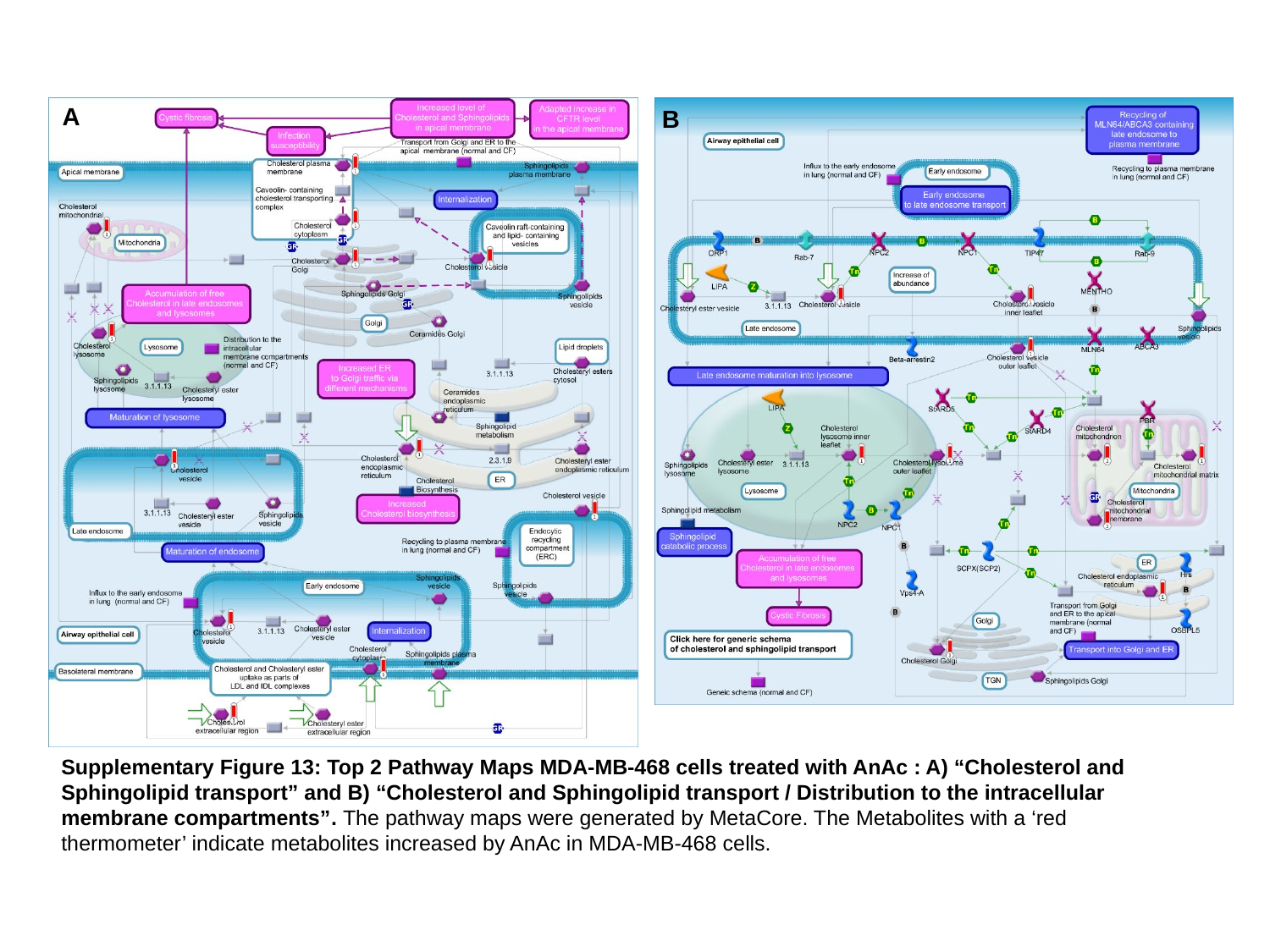

A
B
Supplementary Figure 13: Top 2 Pathway Maps MDA-MB-468 cells treated with AnAc : A) “Cholesterol and Sphingolipid transport” and B) “Cholesterol and Sphingolipid transport / Distribution to the intracellular membrane compartments”. The pathway maps were generated by MetaCore. The Metabolites with a ‘red thermometer’ indicate metabolites increased by AnAc in MDA-MB-468 cells.

## Slide 18
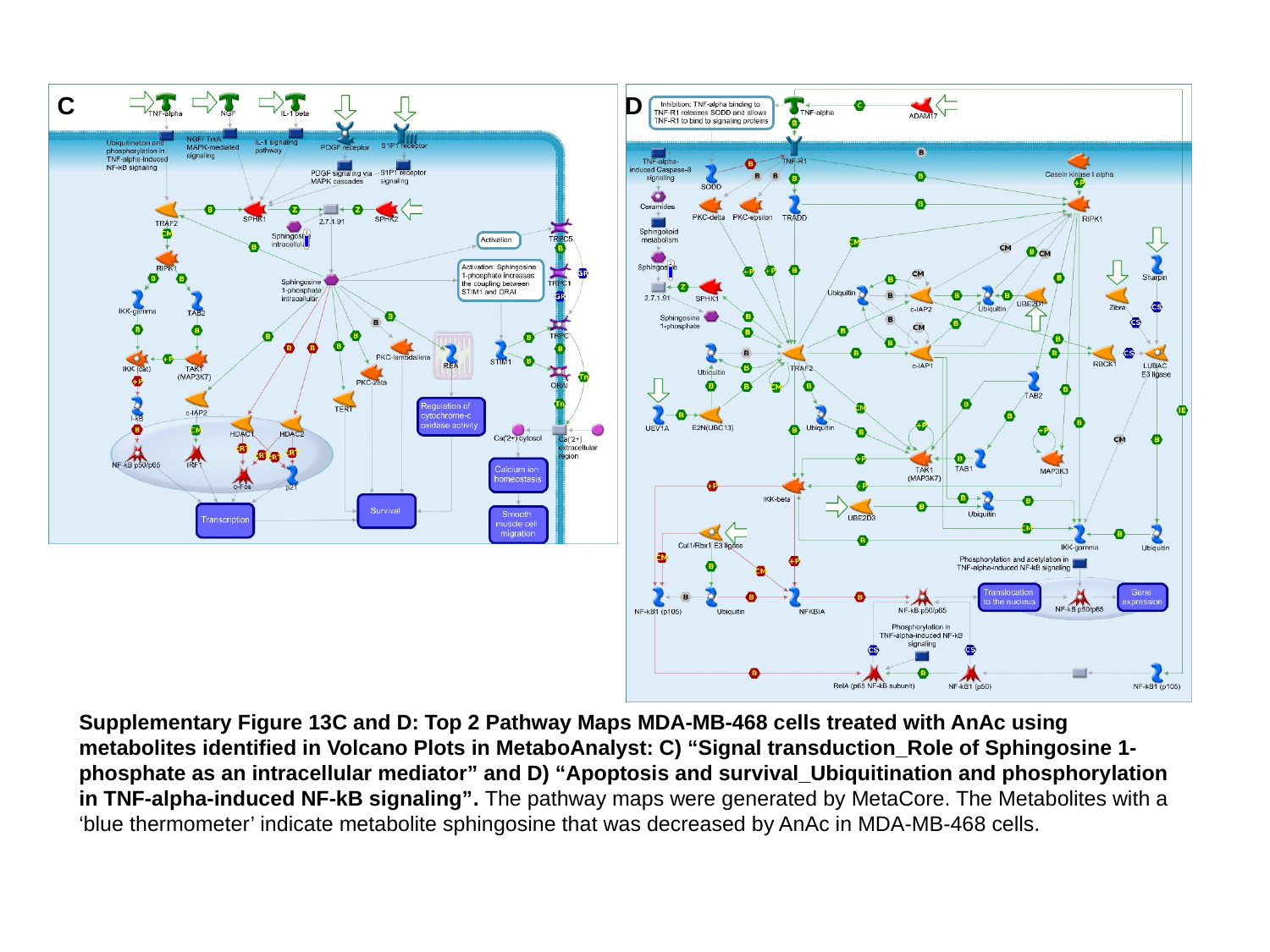

C
D
Supplementary Figure 13C and D: Top 2 Pathway Maps MDA-MB-468 cells treated with AnAc using metabolites identified in Volcano Plots in MetaboAnalyst: C) “Signal transduction_Role of Sphingosine 1-phosphate as an intracellular mediator” and D) “Apoptosis and survival_Ubiquitination and phosphorylation in TNF-alpha-induced NF-kB signaling”. The pathway maps were generated by MetaCore. The Metabolites with a ‘blue thermometer’ indicate metabolite sphingosine that was decreased by AnAc in MDA-MB-468 cells.

## Slide 19
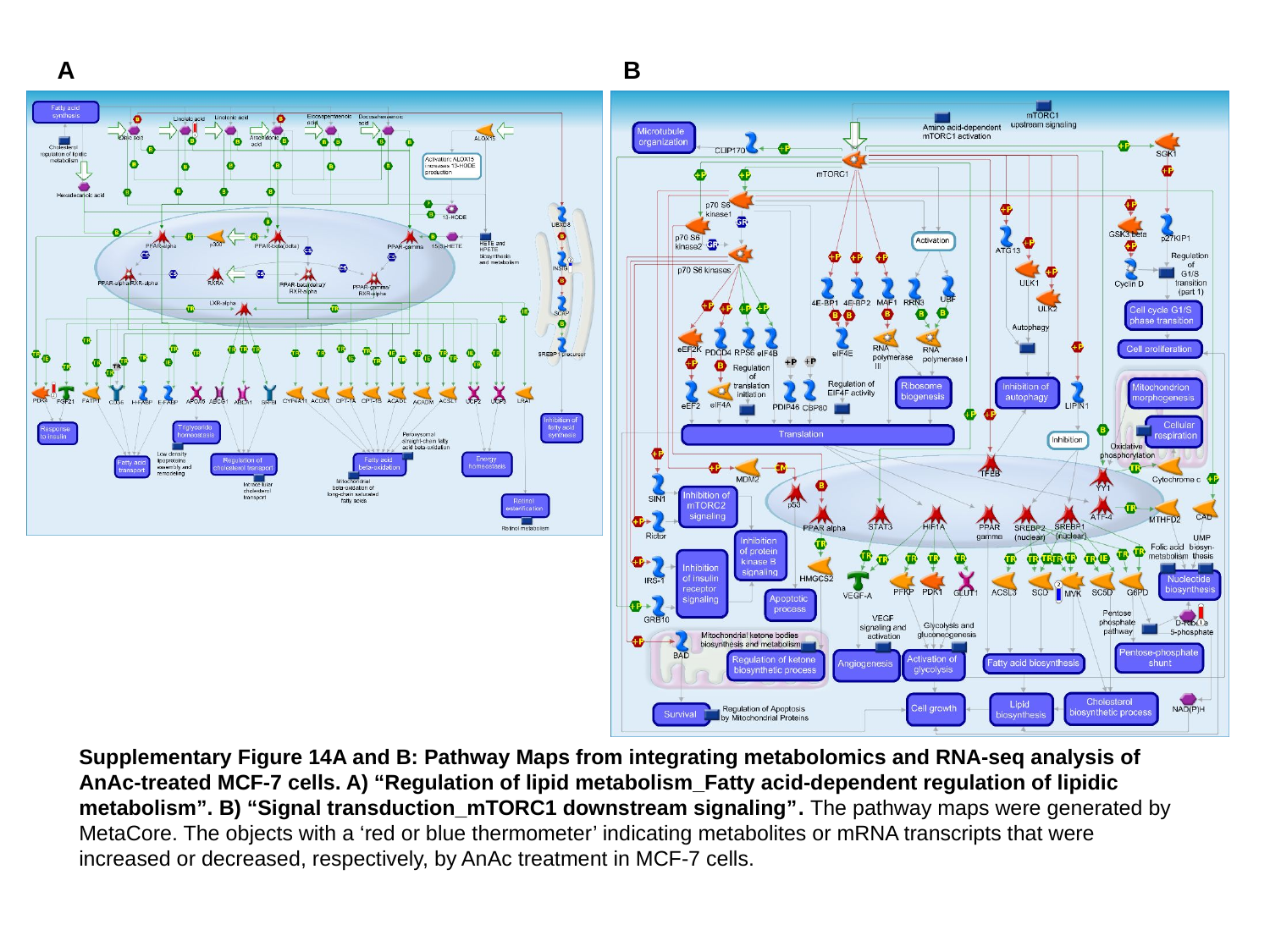

A
B
Supplementary Figure 14A and B: Pathway Maps from integrating metabolomics and RNA-seq analysis of AnAc-treated MCF-7 cells. A) “Regulation of lipid metabolism_Fatty acid-dependent regulation of lipidic metabolism”. B) “Signal transduction_mTORC1 downstream signaling”. The pathway maps were generated by MetaCore. The objects with a ‘red or blue thermometer’ indicating metabolites or mRNA transcripts that were increased or decreased, respectively, by AnAc treatment in MCF-7 cells.

## Slide 20
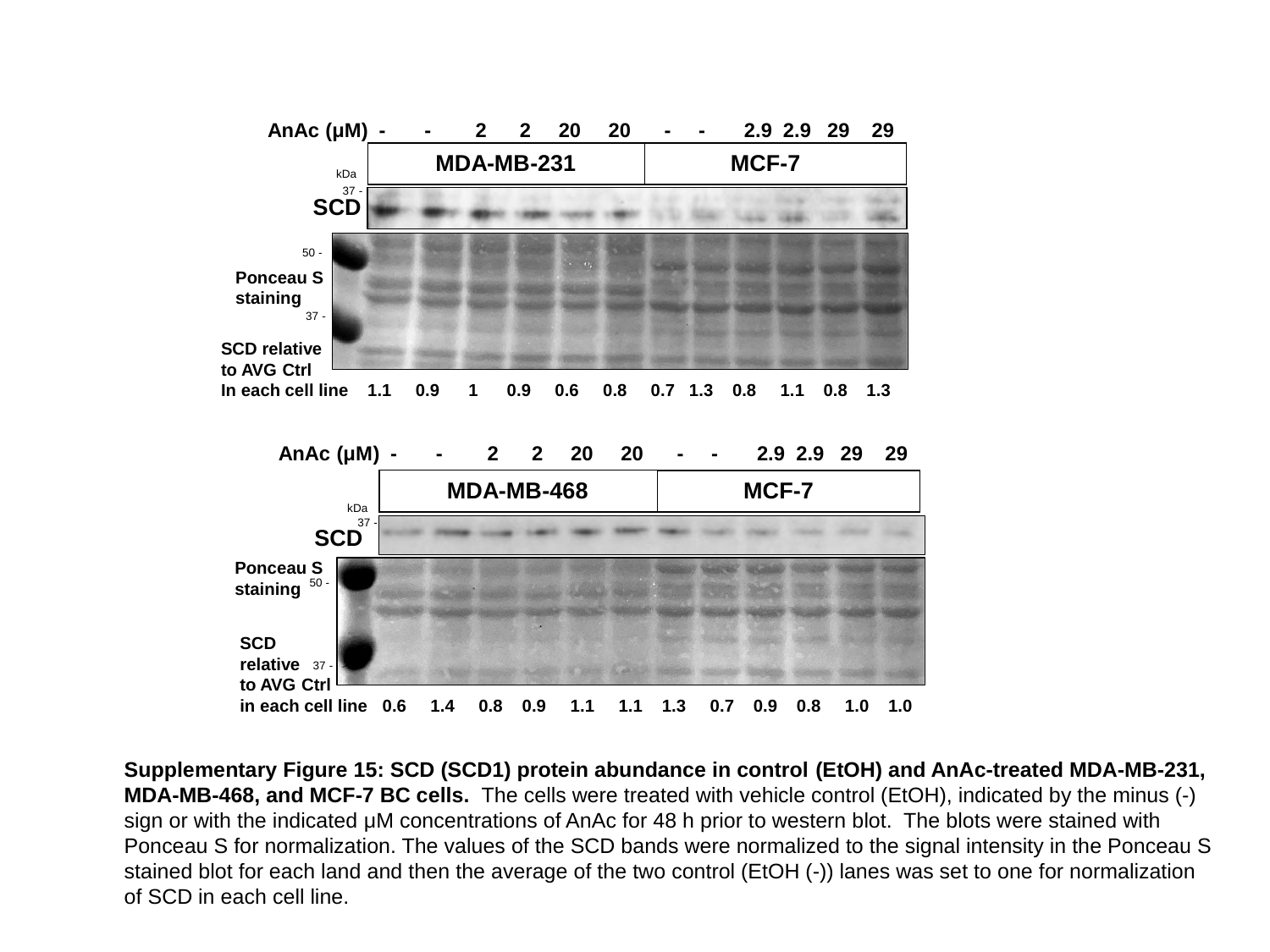

Supplementary Figure 15: SCD (SCD1) protein abundance in control (EtOH) and AnAc-treated MDA-MB-231, MDA-MB-468, and MCF-7 BC cells. The cells were treated with vehicle control (EtOH), indicated by the minus (-) sign or with the indicated μM concentrations of AnAc for 48 h prior to western blot. The blots were stained with Ponceau S for normalization. The values of the SCD bands were normalized to the signal intensity in the Ponceau S stained blot for each land and then the average of the two control (EtOH (-)) lanes was set to one for normalization of SCD in each cell line.

## Slide 21
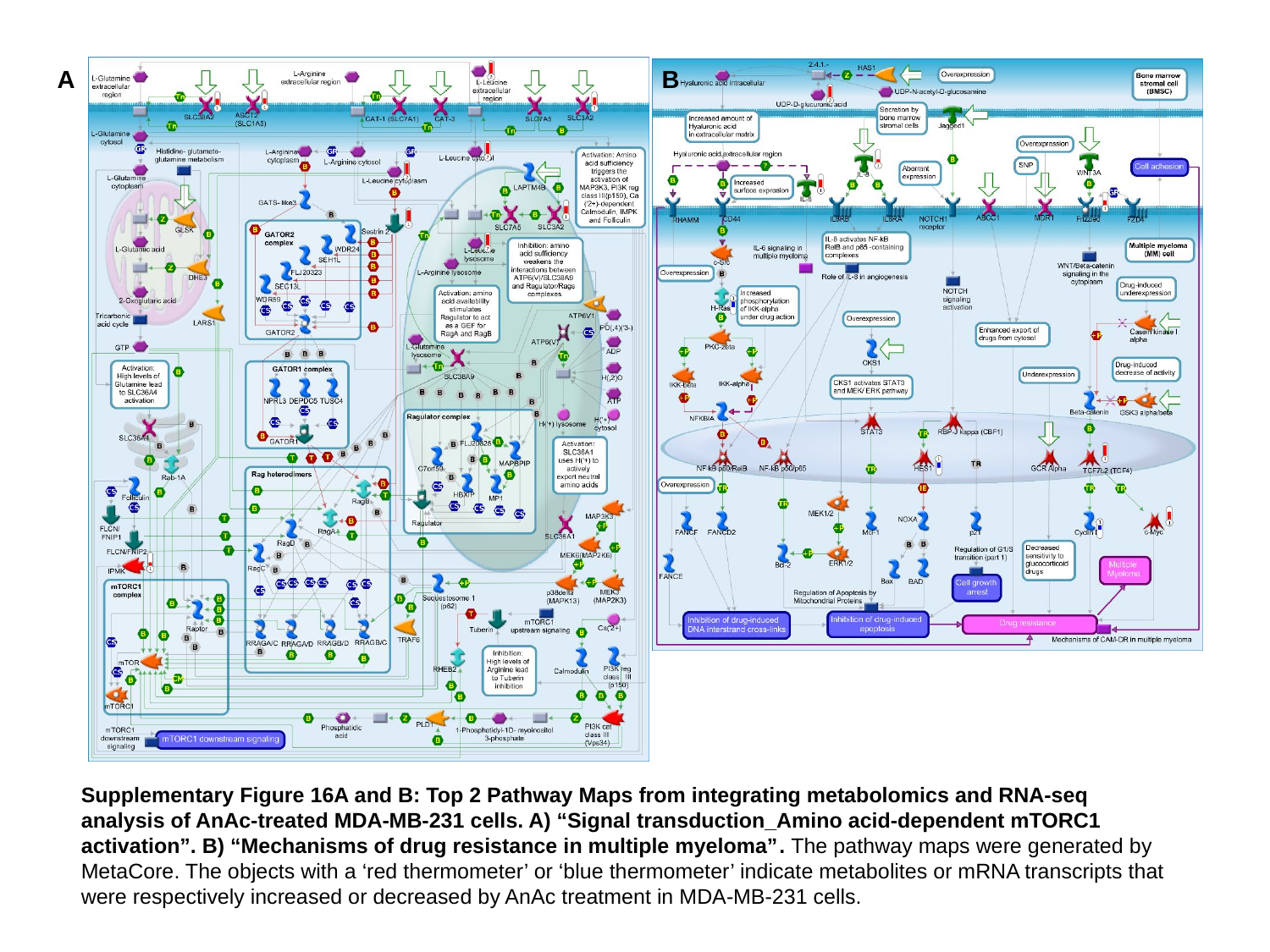

A
B
Supplementary Figure 16A and B: Top 2 Pathway Maps from integrating metabolomics and RNA-seq analysis of AnAc-treated MDA-MB-231 cells. A) “Signal transduction_Amino acid-dependent mTORC1 activation”. B) “Mechanisms of drug resistance in multiple myeloma”. The pathway maps were generated by MetaCore. The objects with a ‘red thermometer’ or ‘blue thermometer’ indicate metabolites or mRNA transcripts that were respectively increased or decreased by AnAc treatment in MDA-MB-231 cells.

## Slide 22
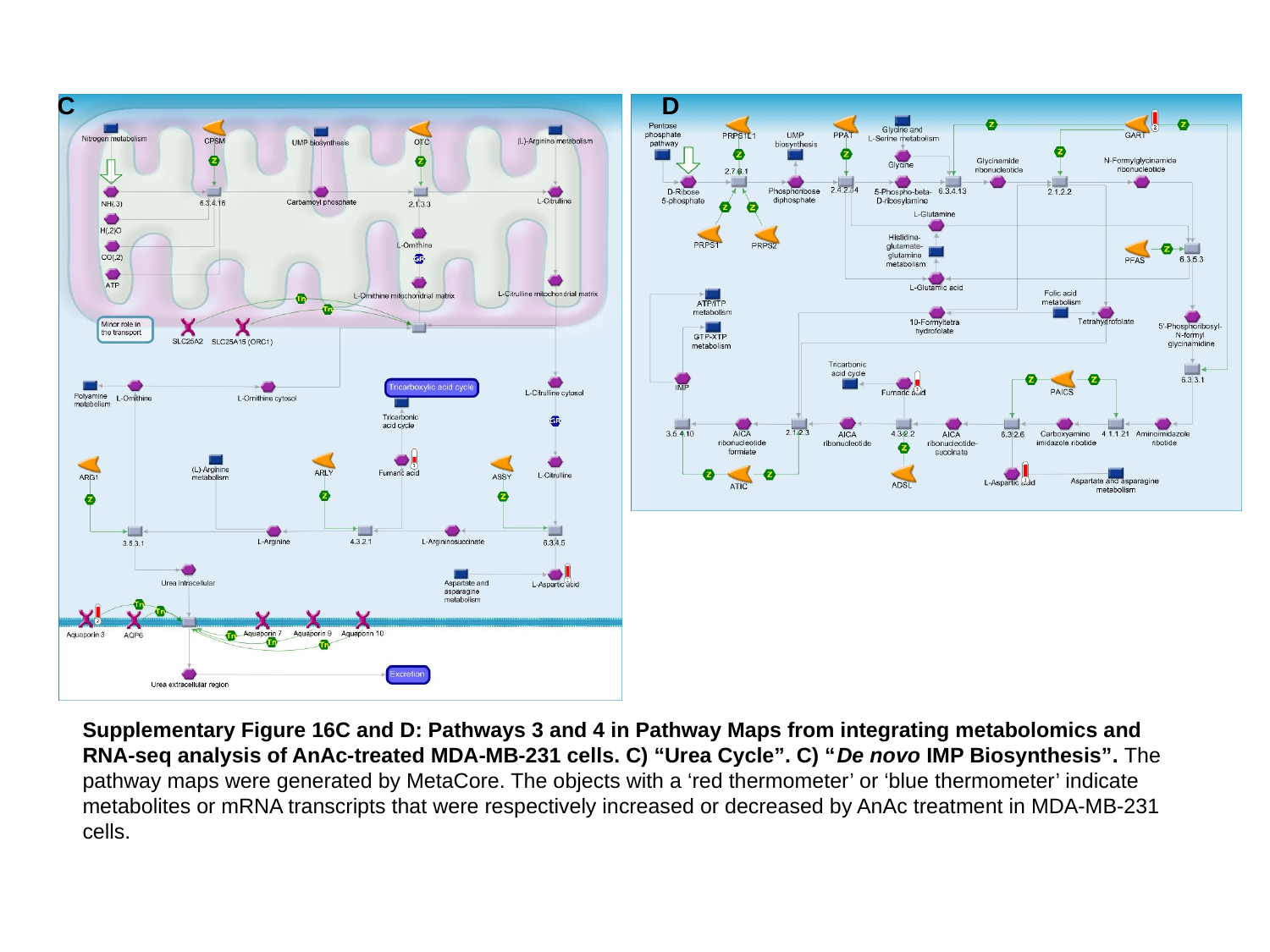

C
D
Supplementary Figure 16C and D: Pathways 3 and 4 in Pathway Maps from integrating metabolomics and RNA-seq analysis of AnAc-treated MDA-MB-231 cells. C) “Urea Cycle”. C) “De novo IMP Biosynthesis”. The pathway maps were generated by MetaCore. The objects with a ‘red thermometer’ or ‘blue thermometer’ indicate metabolites or mRNA transcripts that were respectively increased or decreased by AnAc treatment in MDA-MB-231 cells.

## Slide 23
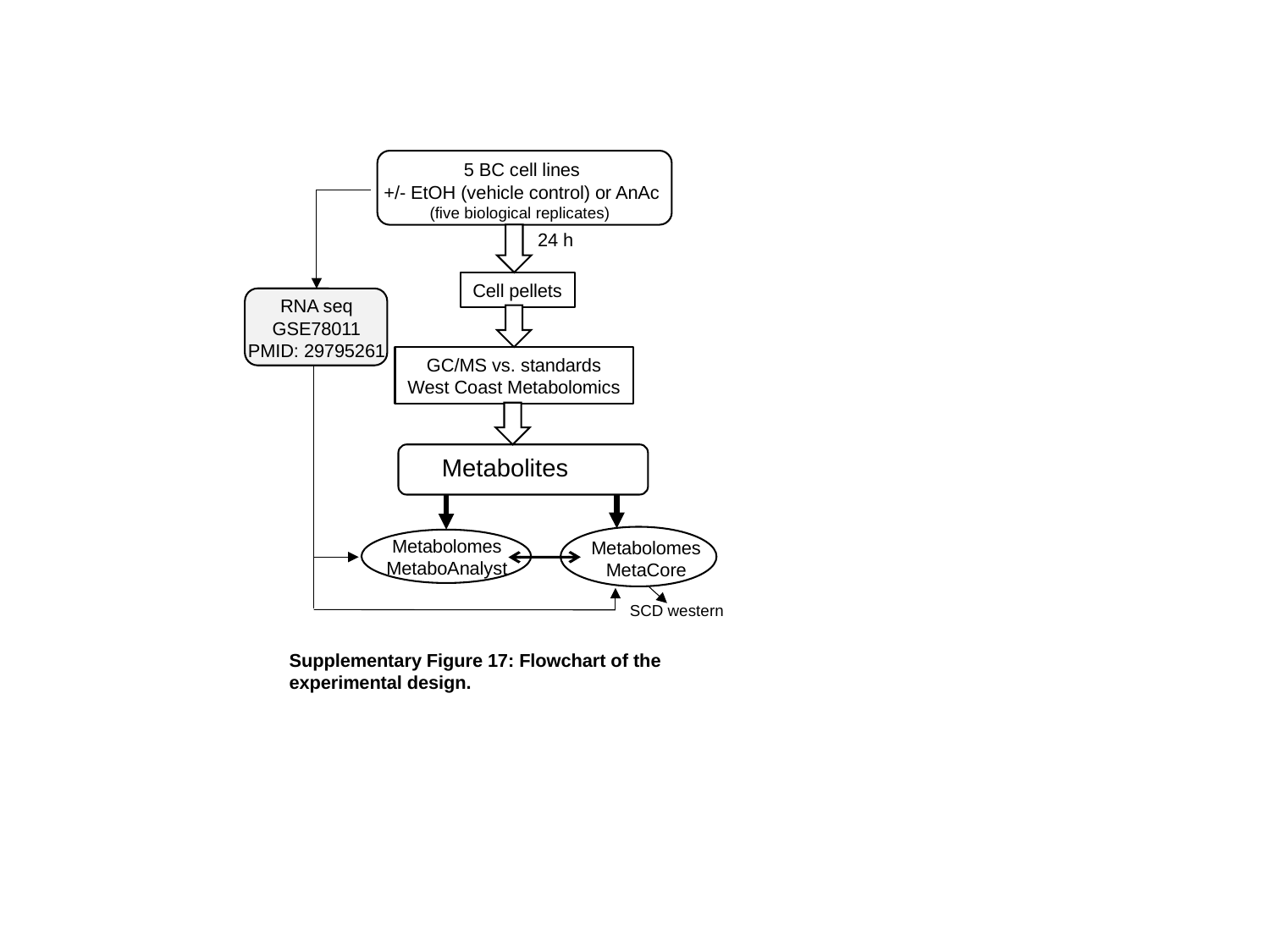

5 BC cell lines
+/- EtOH (vehicle control) or AnAc
(five biological replicates)
24 h
Cell pellets
RNA seq
GSE78011
PMID: 29795261
GC/MS vs. standards
West Coast Metabolomics
Metabolites
Metabolomes
MetaboAnalyst
Metabolomes
MetaCore
SCD western
Supplementary Figure 17: Flowchart of the experimental design.
