# Supplementary material for: Integrated Metabolomics and Transcriptomics Analysis of Anacardic Acid Inhibition of Breast Cancer Cell Viability
Source: Int J Mol Sci. 2024 Jun 27;25(13):7044. doi: 10.3390/ijms25137044 (PMC11241071; doi:10.3390/ijms25137044)
Supplement: Supplementary file 1 [file ijms-25-07044-s001.zip › ijms-3005065-Supplementary Table 2.pdf]

Supplementary Table 2: SAM (significance of metabolites) analysis identified 96 metabolites in EtOH-treated cells. The raw p-value (rawp) represents the probability of observing a test statistic as extreme as the one obtained (or more extreme) under the null hypothesis. It quantifies the evidence against the null hypothesis. Smaller raw p-values indicate stronger evidence against the null hypothesis.

Metabolite	d.value	stdev	rawp	q.value
sorbitol	13.172	0.039969	0	0
hexadecylglycerol	9.3115	0.15814	0	0
octadecylglycerol	8.8032	0.20075	0	0
xanthine	7.0562	0.20849	0	0
ribulose-5-phosphate	6.1624	0.15514	0	0
gluconic acid	5.8991	0.049243	0	0
glucose-6-phosphate	5.5961	0.11415	0	0
xylonic acid	5.3744	0.042004	0	0
1-hexadecanol	5.3732	0.13087	0	0
uracil	5.3582	0.11391	0	0
octadecanol	5.3181	0.11276	0	0
adenine	5.2562	0.036999	0	0
linoleic acid	5.1966	0.075876	0	0
fructose-6-phosphate	4.9937	0.094625	0	0
adenosine-5-monophosphate	4.9072	0.060088	0	0
glucose	4.8487	0.10231	0	0
3-phosphoglycerate	4.577	0.24483	0	0
N-acetylaspartic acid	4.5497	0.17511	0	0
beta-alanine	4.4862	0.14329	0	0
orotic acid	4.394	0.066258	5.18E-05	1.98E-05
ribose-5-phosphate	4.3144	0.19729	5.18E-05	1.98E-05
fructose-1-phosphate	4.2022	0.13056	0.000104	2.52E-05
cystine	4.0354	0.051074	0.000104	2.52E-05
fructose	4.0269	0.077602	0.000104	2.52E-05
ribitol	4.0244	0.047314	0.000104	2.52E-05
glucuronic acid	3.9659	0.070875	0.000104	2.52E-05
pantothenic acid	3.9539	0.091051	0.000104	2.52E-05
UDP-N-acetylglucosamine	3.8259	0.069008	0.000104	2.52E-05
D-erythro-sphingosine	3.7672	0.068053	0.000104	2.52E-05
tocopherol alpha-	3.7162	0.075355	0.000104	2.52E-05
pseudo uridine	3.6337	0.10093	0.000104	2.52E-05
5'-deoxy-5'-methylthioadenosine	3.6114	0.099242	0.000104	2.52E-05
pyruvic acid	3.4391	0.16515	0.000104	2.52E-05
lactamide	3.3185	0.11375	0.000207	3.85E-05
cholesterone	3.2725	0.099211	0.000207	3.85E-05

inosine 5'-monophosphate	3.2337	0.15939	0.000207	3.85E-05
malic acid	3.1588	0.049623	0.000207	3.85E-05
pyrophosphate	3.1208	0.10638	0.000207	3.85E-05
azelaic acid	3.046	0.21347	0.000207	3.85E-05
xylulose	3.0244	0.13758	0.000259	3.85E-05
proline	3.0196	0.16615	0.000259	3.85E-05
2-monopalmitin	3.0021	0.13712	0.000259	3.85E-05
thymine	2.9763	0.11264	0.000259	3.85E-05
palatinitol	2.9077	0.11665	0.000259	3.85E-05
ribose	2.9059	0.14423	0.000259	3.85E-05
glutamine	2.9048	0.10332	0.000259	3.85E-05
methionine	2.9033	0.060519	0.000259	3.85E-05
uridine	2.8426	0.13643	0.000259	3.85E-05
cystathionine	2.8336	0.21514	0.000259	3.85E-05
1,3-diaminopropane	2.8329	0.10589	0.000259	3.85E-05
alpha-aminoadipic acid	2.8135	0.10032	0.000259	3.85E-05
xylitol	2.8123	0.1196	0.000259	3.85E-05
aminomalonate	2.7435	0.18362	0.000259	3.85E-05
maltotriose	2.6886	0.27071	0.000259	3.85E-05
galactinol	2.6817	0.1283	0.000311	4.53E-05
N-carbamoylaspartate	2.6314	0.14791	0.000363	5.19E-05
maleimide	2.6008	0.16986	0.000466	6.56E-05
succinic acid	2.5404	0.18083	0.000518	7.16E-05
aspartic acid	2.5003	0.13576	0.00057	7.74E-05
2-monoolein	2.4909	0.35362	0.000622	8.31E-05
glycerol-alpha-phosphate	2.4783	0.095646	0.000674	8.31E-05
dehydrocholecalciferol	2.4777	0.15052	0.000674	8.31E-05
glycolic acid	2.4605	0.14998	0.000674	8.31E-05
4-hydroxybutyric acid	2.4527	0.11571	0.000674	8.31E-05
threonine	2.4522	0.064165	0.000674	8.31E-05
creatinine	2.4147	0.3307	0.000777	9.30E-05
ornithine	2.4068	0.090938	0.000777	9.30E-05
isothreonic acid	2.3795	0.202	0.000829	9.77E-05
phosphoenolpyruvate	2.3281	0.34236	0.000881	0.000102
cysteine-glycine	2.3122	0.28749	0.000933	0.000104
citric acid	2.3068	0.096364	0.000933	0.000104
lactic acid	2.2988	0.38918	0.000933	0.000104
1-monopalmitin	2.2978	0.10098	0.000984	0.000108
4-aminobutyric acid	2.2706	0.2282	0.001036	0.000112
adenosine	2.2478	0.14834	0.00114	0.000122
UDP-glucuronic acid	2.2232	0.075886	0.001244	0.000131

guanine	2.204	0.20776	0.001347	0.000138
guanosine	2.2016	0.21145	0.001347	0.000138
asparagine	2.1942	0.13272	0.001399	0.000142
lactulose	2.1677	0.42868	0.001658	0.000164
phosphoethanolamine	2.1563	0.13138	0.001658	0.000164
glycine	2.1353	0.094593	0.00171	0.000167
arachidonic acid	2.0927	0.19357	0.001865	0.00018
oleic acid	2.0728	0.22468	0.002021	0.000193
putrescine	2.0075	0.2067	0.002487	0.000235
cholesterol	1.9798	0.13417	0.002643	0.000246
phytosphingosine	1.9252	0.06063	0.003212	0.000296
2,5-dihydroxypyrazine	1.9153	0.24734	0.003316	0.000302
5-aminovaleric acid	1.8945	0.084286	0.003523	0.000304
lignoceric acid	1.8941	0.18713	0.003523	0.000304
alpha-ketoglutarate	1.8934	0.16667	0.003523	0.000304
glyceric acid	1.8866	0.22947	0.003523	0.000304
lysine	1.8837	0.13326	0.003523	0.000304
myo-inositol	1.8705	0.14234	0.003575	0.000305
2-ketoisocaproic acid	1.8349	0.18608	0.003886	0.000328
alanine	1.7674	0.14367	0.004819	0.000402
