# Supplementary material for: Integrated Metabolomics and Transcriptomics Analysis of Anacardic Acid Inhibition of Breast Cancer Cell Viability
Source: Int J Mol Sci. 2024 Jun 27;25(13):7044. doi: 10.3390/ijms25137044 (PMC11241071; doi:10.3390/ijms25137044)
Supplement: Supplementary file 1 [file ijms-25-07044-s001.zip › ijms-3005065-Supplementary Table 3.pdf]

Supplementary Table 3: SAM (significance of metabolites) analysis identified 128 metabolites in AnAc-treated cells. The raw p-value (rawp) represents the probability of observing a test statistic as extreme as the one obtained (or more extreme) under the null hypothesis. It quantifies the evidence against the null hypothesis. Smaller raw p-values indicate stronger evidence against the null hypothesis.

	d.value	stdev	rawp	q.value
sorbitol	14.45	0.026276	0	0
hexadecylglycerol	13.065	0.054233	0	0
octadecylglycerol	12.285	0.072181	0	0
ribulose-5-phosphate	8.7804	0.08786	0	0
xanthine	8.4429	0.192	0	0
uracil	7.8555	0.060745	0	0
linoleic acid	7.0267	0.051386	0	0
ribose-5-phosphate	6.4979	0.11122	0	0
adenine	6.3553	0.038166	0	0
glucuronic acid	5.6074	0.027959	0	0
gluconic acid	5.5565	0.045617	0	0
octadecanol	5.4818	0.20234	0	0
xylonic acid	5.4679	0.035467	0	0
fructose	5.4313	0.036034	0	0
N-acetylaspartic acid	5.4187	0.219	0	0
3-phosphoglycerate	5.4102	0.23337	0	0
galactinol	5.2842	0.12768	0	0
adenosine-5-monophosphate	5.2664	0.091686	0	0
5'-deoxy-5'-methylthioadenosine	5.2602	0.078685	0	0
orotic acid	5.1707	0.075605	0	0
beta-alanine	5.1292	0.17466	0	0
lactic acid	5.0597	0.12993	0	0
pantothenic acid	5.0367	0.10789	0	0
fructose-1-phosphate	4.9707	0.071139	0	0
1-hexadecanol	4.8386	0.25223	5.18E-05	1.39E-05
2-monoolein	4.8256	0.1044	5.18E-05	1.39E-05
cystathionine	4.7984	0.14524	5.18E-05	1.39E-05
thymine	4.7358	0.054424	5.18E-05	1.39E-05
ribitol	4.7046	0.087916	5.18E-05	1.39E-05
cholesterone	4.5649	0.094161	5.18E-05	1.39E-05
creatinine	4.4509	0.25114	5.18E-05	1.39E-05
glucose-6-phosphate	4.3818	0.22491	5.18E-05	1.39E-05
pseudo uridine	4.3748	0.038004	5.18E-05	1.39E-05
alpha-aminoadipic acid	4.2206	0.099717	5.18E-05	1.39E-05
xylulose	4.1777	0.034653	5.18E-05	1.39E-05

ribose	4.1517	0.052047	5.18E-05	1.39E-05
docosahexaenoic acid	4.1251	0.081914	5.18E-05	1.39E-05
arachidonic acid	4.1202	0.1161	5.18E-05	1.39E-05
lactamide	4.0912	0.10441	5.18E-05	1.39E-05
fructose-6-phosphate	4.068	0.21647	5.18E-05	1.39E-05
aspartic acid	4.0205	0.079361	5.18E-05	1.39E-05
tocopherol alpha-	4.0079	0.091979	5.18E-05	1.39E-05
cysteine	3.9842	0.24717	5.18E-05	1.39E-05
UDP-N-acetylglucosamine	3.7701	0.084656	0.000104	2.72E-05
malic acid	3.6922	0.04983	0.000155	3.90E-05
cysteine-glycine	3.6874	0.18011	0.000155	3.90E-05
D-erythro-sphingosine	3.6214	0.066061	0.000311	7.14E-05
isothreonic acid	3.6126	0.18674	0.000311	7.14E-05
proline	3.5849	0.14825	0.000311	7.14E-05
glutamine	3.5048	0.086021	0.000363	7.14E-05
adenosine	3.49	0.07	0.000363	7.14E-05
ornithine	3.464	0.079769	0.000363	7.14E-05
1-monoolein	3.3323	0.19132	0.000363	7.14E-05
cystine	3.3262	0.14845	0.000363	7.14E-05
succinic acid	3.3165	0.16714	0.000363	7.14E-05
uridine	3.2589	0.083961	0.000415	7.14E-05
myristic acid	3.2444	0.074381	0.000415	7.14E-05
N-carbamoylaspartate	3.2066	0.15571	0.000415	7.14E-05
glycolic acid	3.205	0.15806	0.000415	7.14E-05
pyrophosphate	3.1846	0.1236	0.000415	7.14E-05
N-acetylglucosamine	3.1165	0.077183	0.000415	7.14E-05
1,3-diaminopropane	3.1076	0.062926	0.000415	7.14E-05
methionine	3.0779	0.053375	0.000415	7.14E-05
lysine	3.0433	0.089979	0.000415	7.14E-05
maleimide	3.0373	0.20752	0.000415	7.14E-05
UDP-glucuronic acid	3.0004	0.12272	0.000415	7.14E-05
pyruvic acid	2.9766	0.21544	0.000415	7.14E-05
glycerol-alpha-phosphate	2.947	0.12708	0.000518	8.54E-05
aminomalonate	2.9304	0.18553	0.000518	8.54E-05
lactulose	2.9251	0.36342	0.000518	8.54E-05
glyceric acid	2.923	0.19423	0.00057	9.26E-05
xylitol	2.8861	0.084396	0.000622	9.96E-05
maltotriose	2.8197	0.24203	0.000674	0.000105
spermine	2.8087	0.22752	0.000674	0.000105
pentadecanoic acid	2.7907	0.10618	0.000725	0.00011
glucose	2.7766	0.22999	0.000725	0.00011

cholesterol	2.7587	0.093364	0.000777	0.000113
guanine	2.7528	0.10046	0.000777	0.000113
inosine 5'-monophosphate	2.7188	0.29837	0.000777	0.000113
heptadecanoic acid	2.6592	0.12936	0.000829	0.000117
hydroxycarbamate	2.6585	0.20113	0.000829	0.000117
phosphoethanolamine	2.6285	0.10858	0.000829	0.000117
ethanol phosphate	2.5562	0.096681	0.000881	0.00012
azelaic acid	2.5161	0.19152	0.000881	0.00012
4-hydroxybutyric acid	2.5161	0.090575	0.000881	0.00012
phosphate	2.5033	0.23427	0.000984	0.000131
glutathione	2.4974	0.12293	0.000984	0.000131
monomyristin	2.4828	0.052813	0.001036	0.000136
dehydroabietic acid	2.4324	0.16177	0.001295	0.000164
1-monopalmitin	2.4192	0.081404	0.001295	0.000164
lactose	2.4127	0.20173	0.001295	0.000164
2-ketoisocaproic acid	2.3857	0.19385	0.001503	0.000188
cis-gondoic acid	2.3772	0.12862	0.001554	0.000193
threonine	2.3543	0.099947	0.001658	0.000203
4-aminobutyric acid	2.3436	0.31359	0.001762	0.000214
adipic acid	2.3191	0.15576	0.001814	0.000216
palmitic acid	2.3161	0.13511	0.001814	0.000216
glycine	2.2818	0.092329	0.001969	0.000229
asparagine	2.2795	0.18851	0.001969	0.000229
stearic acid	2.2722	0.14503	0.002073	0.000239
ile-ile	2.2119	0.14538	0.002435	0.000275
lanosterol	2.2115	0.22438	0.002435	0.000275
citric acid	2.1997	0.14224	0.002487	0.000276
palatinitol	2.1975	0.17146	0.002487	0.000276
nonadecanoic acid	2.1704	0.11221	0.002694	0.000293
guanosine	2.1697	0.1132	0.002694	0.000293
xanthosine	2.1268	0.091687	0.003109	0.000335
2,5-dihydroxypyrazine	2.1184	0.28775	0.003161	0.000338
2-ketoadipic acid	2.0953	0.16577	0.003316	0.000351
5-aminovaleric acid	2.0739	0.11079	0.003523	0.000369
hypoxanthine	2.0438	0.13241	0.003834	0.000395
salicylic acid	2.0434	0.22814	0.003834	0.000395
putrescine	2.0113	0.32819	0.004352	0.000431
serotonin	2.0098	0.12047	0.004352	0.000431
2-hydroxyvaleric acid	2.0026	0.10212	0.004352	0.000431
histidine	1.9963	0.25795	0.004404	0.000431
phytosphingosine	1.9896	0.080696	0.004404	0.000431

nicotinamide	1.9867	0.16455	0.004404	0.000431
benzoic acid	1.9837	0.14248	0.004456	0.000432
glucose-1-phosphate	1.9611	0.12381	0.004871	0.000468
2-hydroxyglutaric acid	1.9418	0.086131	0.00513	0.000485
capric acid	1.9411	0.15113	0.00513	0.000485
lauric acid	1.9124	0.12172	0.005596	0.000525
sinigrin	1.8678	0.12264	0.006062	0.000564
arabitol	1.8653	0.18302	0.006166	0.000569
dodecanol	1.842	0.12295	0.00658	0.000602
cytidine-5-monophosphate	1.8194	0.10986	0.006839	0.000621
ethanolamine	1.8022	0.18584	0.007202	0.000649
