# Supplementary material for: Integrated Metabolomics and Transcriptomics Analysis of Anacardic Acid Inhibition of Breast Cancer Cell Viability
Source: Int J Mol Sci. 2024 Jun 27;25(13):7044. doi: 10.3390/ijms25137044 (PMC11241071; doi:10.3390/ijms25137044)
Supplement: Supplementary file 1 [file ijms-25-07044-s001.zip › ijms-3005065-Supplementary Tables S4-S8.pdf]

Supplementary Table 4A: Top 10 enrichment by pathway maps for metabolites identified as significantly altered by AnAc (versus EtOH) in MCF-7 cells in MetaCore.

Pathway Maps	P Value	metabolites
Signal transduction: Amino acid-dependent mTORC1 activation	1.495E-06	Leucine, 2-Oxoglutaric acid (alpha-ketoglutarate)
Glycine and L-Serine metabolism	7.455E-06	Glycine, 2-Oxoglutaric acid (alpha-ketoglutarate)
Metabolism in pancreatic cancer cells	1.028E-05	2-Oxoglutaric acid (alpha-ketoglutarate), D-Ribose 5-phosphate , Glycine, D-Ribulose-5-phosphate
Immune response; Distinct metabolic pathways in naive and effector CD8+ T cells	1.622E-05	Leucine, 2-Oxoglutaric acid (alpha-ketoglutarate), D-Ribose 5-phosphate
Pyruvate metabolism	3.496E-04	Phosphoenolpyruvate
Immune response: The effect of IDO1 on T cell metabolism	8.402E-04	2-Oxoglutaric acid (alpha-ketoglutarate), Phosphoenolpyruvate, D-Ribose 5-phosphate
Pentose phosphate pathway	2.305E-03	D-Ribose 5-phosphate, D-Ribulose-5-phosphate
Beta-alanine metabolism	2.441E-03	2-Oxoglutaric acid(alpha-ketoglutarate), Uracil
Nitrogen metabolism	2.582E-03	2-Oxoglutaric acid (alpha-ketoglutarate), Glycine
De novo IMP biosynthesis	3.025E-03	D-Ribose 5-phosphate, Glycine

Supplementary Table 4B: Top 10 pathway maps for metabolites identified as significantly altered by AnAc (versus EtOH) in MCF-7 cells in MetaboAnalyst Volcano Plots.

Pathway Maps	P Value	metabolites
Immune response: The effect of IDO1 on T cell metabolism	5.636E-06	D-Ribose 5-phosphate, beta-D-Glucose, Phosphoenolpyruvate, beta-D-Fructose 6-phosphate
Pentose phosphate pathway	1.272E-05	D-Ribose 5-phosphate, beta-D-Fructose 6-phosphate, D-Ribulose-5-phosphate
Pyruvate metabolism	9.464E-05	Phosphoenolpyruvate
Metabolism in pancreatic cancer cells	9.464E-05	D-Ribose 5-phosphate, beta-D-Fructose 6-phosphate, D-Ribulose-5-phosphate
Fructose metabolism	1.742E-04	beta-D-Glucose, D-Fructose-1-phosphate, beta-D-Fructose 6-phosphate
Glycolysis and gluconeogenesis	2.707E-04	beta-D-Glucose intracellular, Phosphoenolpyruvate cytoplasm, beta-D-Fructose 6-phosphate intracellular
Putative pathways of n-3 and n-6 fatty acids in obesity and type 2 diabetes	2.196E-03	Docosahexaenoic acid (DHA)
Amino sugar metabolism	3.026E-03	Phosphoenolpyruvate, beta-D-Fructose 6-phosphate
TTP metabolism	3.870E-03	Thymine, Uracil
CTP/UTP metabolism	9.334E-03	Cytidine, Uracil

Supplementary Table 5A: Top 10 enrichment by pathway maps for metabolites identified as significantly altered by AnAc (versus EtOH) in HCC1806 cells in MetaCore.

Pathway Maps	P Value	metabolites
Glycogen metabolism	8.948E-05	Maltotriose, alpha-D-Glucose 1-phosphate
Transport_GPR40 signaling pathway in beta cells	1.917E-02	UDP-N-acetyl-D-glucosamine
Mechanisms of drug resistance in multiple myeloma	1.998E-02	UDP-N-acetyl-D-glucosamine
Immune response_IL-13 signaling via PI3K-ERK pathway	2.038E-02	Putrescine
Gamma-aminobutyrate (GABA) biosynthesis and metabolism	2.119E-02	Putrescine
Influence of bone marrow cell environment on progression of multiple myeloma	2.119E-02	UDP-N-acetyl-D-glucosamine
Amino sugar metabolism	2.442E-02	UDP-N-acetyl-D-glucosamine
Galactose metabolism	2.563E-02	alpha-D-Glucose 1-phosphate
Polyamine metabolism	2.603E-02	Putrescine
L-Arginine metabolism	3.205E-02	Putrescine

Supplementary Table 5B: Six pathway maps for metabolites identified as significantly altered by AnAc (versus EtOH) in HCC1806 cells in MetaboAnalyst Volcano Plot as identified in MetaCore.

Pathway Maps	P Value	metabolites
Glycogen metabolism	2.628E-05	Maltotriose, alpha-D-Glucose 1-phosphate
Immune response_IL-13 signaling via PI3K-ERK pathway	1.213E-02	Putrescine
Gamma-aminobutyrate (GABA) biosynthesis and metabolism	1.261E-02	Putrescine
Galactose metabolism	1.526E-02	alpha-D-Glucose 1-phosphate
Polyamine metabolism	1.550E-02	Putrescine
L-Arginine metabolism	1.912E-02	Putrescine

Supplementary Table 6A: Top 10 enrichment by pathway maps for metabolites identified as significantly altered by AnAc (versus EtOH) in BT-20 cells in MetaCore.

Pathway Maps	P Value	metabolites
Immune response_Distinct metabolic pathways in naive and effector CD8+ T cells	1.557E-06	Citric acid, L-Glutamine
Immune response_The effect of IDO1 on T cell metabolism	3.269E-06	Citric acid, L-Glutamine
Signal transduction_Amino acid-dependent mTORC1 activation	4.425E-06	L-Glutamine
Neurophysiological process_GABAergic neurotransmission	2.936E-05	L-Glutamine
Metabolism in pancreatic cancer cells	6.381E-05	L-Glutamine
Saturated fatty acids synthesis to hexadecanoic acid	2.265E-04	Citric acid
De novo IMP biosynthesis	9.990E-04	Fumaric acid, L-Glutamine
Immune response_PD-1-induced metabolic changes in T cells	1.574E-03	L-Glutamine
Mechanisms of drug resistance in multiple myeloma	1.574E-03	UDP-N-acetyl-D-glucosamine, UDP-D-glucuronic acid
Influence of bone marrow cell environment on progression of multiple myeloma	1.771E-03	UDP-N-acetyl-D-glucosamine, UDP-D-glucuronic acid

Supplementary Table 6B: Top 10 enrichment by pathway maps for metabolites identified as significantly altered by AnAc (versus EtOH) in BT-20 cells in MetaboAnalyst Volcano Plots (Table 5).

Pathway Maps	P Value	metabolites
Immune response_Distinct metabolic pathways in naive and effector CD8+ T cells	1.953E-08	Citric acid, 2-Oxoglutaric acid (alpha ketoglutarate), L-Glutamine
Immune response: The effect of IDO1 on T cell metabolism	4.982E-08	Citric acid, 2-Oxoglutaric acid (alpha-ketoglutarate), L-Glutamine
Signal transduction_Amino acid-dependent mTORC1 activation	7.302E-08	2-Oxoglutaric acid (alpha-ketoglutarate), L-Glutamine
Neurophysiological process_GABAergic neurotransmission	3.438E-07	2-Oxoglutaric acid (alpha-ketoglutarate), L-Glutamine
Metabolism in pancreatic cancer cells	9.809E-07	2-Oxoglutaric acid (alpha-ketoglutarate), L-Glutamine
Renal secretion of organic electrolytes / Rodent version	1.644E-06	Citric acid, 2-Oxoglutaric acid (alpha-ketoglutarate)
Histidine-glutamate-glutamine metabolism	1.890E-04	2-Oxoglutaric acid (alpha-ketoglutarate), L-Glutamine
Saturated fatty acids synthesis to hexadecanoic acid	2.265E-04	Citric acid
Nitrogen metabolism	8.511E-04	2-Oxoglutaric acid (alpha-ketoglutarate), L-Glutamine
Renal secretion of drugs / Rodent version	1.449E-03	2-Oxoglutaric acid (alpha-ketoglutarate), L-Glutamine

Supplementary Table 7A: Top 10 enrichment by pathway maps for metabolites identified as significantly altered by AnAc (versus EtOH) in MDA-MB-231 cells in MetaCore.

Pathway Maps	P Value	metabolites
Signal transduction_Amino acid-dependent mTORC1 activation	2.465E-06	L-Leucine
Urea cycle	6.506E-04	L-Aspartic acid, Fumaric acid
De novo IMP biosynthesis	7.638E-04	L-Aspartic acid, Fumaric acid
Aspartate and asparagine metabolism	2.654E-03	L-Aspartic acid, Fumaric acid
Immune response_Distinct metabolic pathways in naive and effector CD8+ T cells	2.726E-03	L-Leucine
L-Arginine metabolism	3.100E-03	L-Aspartic acid, Fumaric acid
Aminoacyl-tRNA biosynthesis in mitochondrion	3.256E-03	L-Aspartic acid, L-Leucine
Aminoacyl-tRNA biosynthesis in cytoplasm	4.635E-03	L-Aspartic acid, L-Leucine
Neurophysiological process_Role of CDK5 in presynaptic signaling	2.952E-02	Inositol 4-phosphate
Prolactin signaling in Prostate Cancer	3.471E-02	L-Aspartic acid

Supplementary Table 7B: Top 10 enrichment by pathway maps for metabolites identified as significantly altered by AnAc (versus EtOH) in MDA-MB-231 cells in MetaboAnalyst Volcano Plot 1.5 as identified in MetaCore.

Pathway Maps	P Value	metabolites
Neurophysiological process_Role of CDK5 in presynaptic signaling	1.356E-02	Inositol 4-phosphate
Prolactin signaling in Prostate Cancer	1.597E-02	L-Aspartic acid
Regulation of CFTR gating (normal and CF)	1.645E-02	Pyrophosphate
Beta-alanine metabolism	1.693E-02	L-Aspartic acid
Development_Thrombospondin 1 signaling	1.741E-02	Pyrophosphate
Urea cycle	1.741E-02	L-Aspartic acid
De novo IMP biosynthesis	1.885E-02	L-Aspartic acid
Neurophysiological process_Glucose-inhibited neurons of ventromedial and arcuate nuclei	1.981E-02	Pyrophosphate
Neurophysiological process_Circadian rhythm	2.268E-02	Pyrophosphate
Neurophysiological process_GABAergic neurotransmission	2.459E-02	4-Hydroxybutanoic acid i

Supplementary Table 8A: Top 10 enrichment by pathway maps for metabolites identified as significantly altered by AnAc (versus EtOH) in MDA-MB-468 cells in MetaCore.

Pathway Maps	P Value	metabolites
Apoptosis and survival_Ubiquitination and phosphorylation in TNF-alpha-induced NF-kB signaling	3.166E-03	Sphingosine intracellular
Development_VEGF signaling and activation	3.409E-03	Sphingosine intracellular
Chemotaxis_C5a-induced chemotaxis	3.571E-03	Sphingosine intracellular
IgE- and MGF-induced Fyn-mediated activation of lung mast cells in asthma	3.977E-03	Sphingosine intracellular
Growth factors in regulation of oligodendrocyte precursor cells proliferation in multiple sclerosis	4.383E-03	Sphingosine intracellular
Lysophospholipid mediators-induced inflammatory signaling in normal and asthmatic airway epithelium	4.627E-03	Sphingosine intracellular
Development_Regulation of endothelial progenitor cell differentiation from adult stem cells	4.870E-03	Sphingosine intracellular
Immune response_Fc epsilon RI pathway: signaling through Fyn and PI3K	4.951E-03	Sphingosine intracellular
Immune response_iC3b-induced phagocytosis via alpha-M/beta-2 integrin	4.951E-03	Sphingosine intracellular
Immune response_IL-1 signaling pathway	6.656E-03	Sphingosine intracellular

Supplementary Table 8B: Top 10 enrichment by pathway maps for metabolites identified as significantly altered by AnAc (versus EtOH) in MDA-MB-468 cells in MetaboAnalyst Volcano Plot 1.5 as identified in MetaCore.

Pathway Maps	P Value	metabolites
Signal transduction_Role of Sphingosine 1-phosphate as an intracellular mediator	5.452E-03	Sphingosine intracellular
Apoptosis and survival_Ubiquitination and phosphorylation in TNF-alpha-induced NF-kB signaling	5.906E-03	Sphingosine intracellular
Development_VEGF signaling and activation	6.360E-03	Sphingosine intracellular
Chemotaxis_C5a-induced chemotaxis	6.662E-03	Sphingosine intracellular
Transport_GPR40 signaling in beta cells	7.115E-03	N-Acetyl-D-Glucosamine 6-phosphate intracellular
IgE- and MGF-induced Fyn-mediated activation of lung mast cells in asthma	7.417E-03	Sphingosine intracellular
Growth factors in regulation of oligodendrocyte precursor cells proliferation in multiple sclerosis	8.173E-03	Sphingosine intracellular
Lysophospholipid mediators-induced inflammatory signaling in normal and asthmatic airway epithelium	8.626E-03	Sphingosine intracellular
Aminosugar metabolism	9.079E-03	N-Acetyl-D-Glucosamine 6-phosphate intracellular
Development_Regulation of endothelial progenitor cell differentiation from adult stem cells	9.079E-03	Sphingosine intracellular
